# Elevated histone demethylase KDM5C increases recurrent miscarriage risk by preventing trophoblast proliferation and invasion

**DOI:** 10.1038/s41420-022-01284-y

**Published:** 2022-12-22

**Authors:** Min Xiao, Yan Zheng, Meng-Xi Wang, Yi-Hua Sun, Juan Chen, Kang-Yong Zhu, Fan Zhang, Yun-Hui Tang, Fan Yang, Ting Zhou, Yue-Ping Zhang, Cai-Xia Lei, Xiao-Xi Sun, Shan-He Yu, Fu-Ju Tian

**Affiliations:** 1 Shanghai Ji Ai Genetics and IVF Institute, the Obstetrics and Gynecology Hospital of Fudan University, Shanghai, 200011 China; 2 Department of Pancreatic Surgery, Shanghai General Hospital, Shanghai Jiao Tong University School of Medicine, Shanghai, 200080 China; 3 Shanghai Key Laboratory of Pancreatic Disease, Institute of Pancreatic Disease, Shanghai Jiao Tong University School of Medicine, Shanghai, 200080 China; 4 Shanghai Institute of Hematology, State Key Laboratory of Medical Genomics, National Research Center for Translational Medicine at Shanghai, Ruijin Hospital, Shanghai Jiao Tong University School of Medicine, Shanghai, 200011 China; 5 Department of Pathology, the Obstetrics and Gynecology Hospital of Fudan University, Shanghai, 200011 China; 6 Department of Family Planning, the Obstetrics and Gynecology Hospital of Fudan University, Shanghai, 200011 China; 7 Department of Orthodontics, Shanghai Ninth People’s Hospital, Shanghai Jiao Tong University School of Medicine, Shanghai, 200011 China; 8 The International Peace Maternity & Child Health Hospital, Shanghai Jiao Tong University School of Medicine, Shanghai, 200030 China; 9 Shanghai Key Laboratory of Embryo Original Diseases, Shanghai, 200030 China

**Keywords:** Infertility, Endocrine reproductive disorders

## Abstract

KDM5C is a histone H3K4-specific demethylase, which has been shown to play a key role in biological disease and development. However, the role of KDM5C in trophoblasts at early pregnancy is currently unknown. Here, we showed that KDM5C was upregulated in placental trophoblasts from recurrent miscarriage (RM) patients compared with healthy controls (HCs). Trophoblast proliferation and invasion was inhibited by KDM5C overexpression and was promoted by *KDM5C* knockdown. Transcriptome sequencing revealed that elevated KDM5C exerted anti-proliferation and anti-invasion effects by repressing the expression of essential regulatory genes. The combination analysis of RNA-seq, ChIP-seq and CUT&Tag assay showed that KDM5C overexpression leads to the reduction of H3K4me3 on the promoters and the corresponding downregulation of expression of several regulatory genes in trophoblasts. Among these genes, *TGFβ2* and *RAGE* are essential for the proliferation and invasion of trophoblasts. Importantly, overexpression of KDM5C by a systemically delivered KDM5C adenovirus vector (Ad-KDM5C) promoted embryo resorption rate in mouse. Our results support that KDM5C is an important regulator of the trophoblast function during early pregnancy, and suggesting that KDM5C activity could be responsible for epigenetic alterations seen RM disease.

## Introduction

Pregnancy in humans is a complicated and dynamic series of biological processes that require synergistic cooperation between the receptive uterine endometrium and the embryo developing within [1]. Several critical steps are essential for successful embryo implantation, including trophoblast development, fertilization, immune regulation, and proper maternal-fetal crosstalk [2]. During the first trimester of pregnancy, placental development occurs. Mononuclear cytotrophoblasts (CTBs) are identified as participating in the early stages of human placental development [3]. Villous cytotrophoblasts (vCTB) are trophoblast progenitor cells that can differentiate into syncytiotrophoblast (STB). By contrast, decidual cell column trophoblasts (dCCTs) enter endoreduplicative cycles, and undergo polyploidization and senescence upon differentiation into extravillous trophoblasts (EVTs) [3]. Further, EVTs develop in placental anchoring villi, and migrate into the maternal decidual stroma and its vessels as so-called interstitial cytotrophoblasts (iCTBs) and endovascular cytotrophoblasts (eCTB) [4, 5]. During pregnancy, EVTs are critical in the placenta development. A reduction in EVT invasion may cause uteroplacental insufficiency to develop during pregnancy, which can increase the risk of pre-eclampsia and early recurrent miscarriage (RM) [6, 7]. RM is as the occurrence of three or more sequential spontaneous abortions prior to reaching a gestation of twenty weeks. The frequency of RM in women during their child-bearing years is 1–3% [8]. RM presents a continued challenge for patients and their physicians [9]. Therefore, we need to elucidate the molecular mechanisms underlying the pathogenesis of RM to discover potential therapeutic targets.

Epigenetic modifications are known to be crucial in maternal-fetal medicine [10]. Chromatin is a macromolecular complex that mainly comprises DNA, RNA, and histone protein. Epigenetic modifiers are important regulators of gene expression during cell-fate determination and embryonic development [11]. Previous study has been found that alterations to DNA methylation may cause dysfunction in trophoblastic cells and contribute to RM [12]. Yet, the potential for histone modifiers to play a role in RM remains to be determined. Emerging evidence indicates that histone modification, particularly by lysine methyltransferases (‘writer’) and demethylases (‘eraser’), plays a key role in gene expression and regulation [13, 14]. Members of Jumonji-C (JmjC)-domain containing several histone demethylases are engaged in complex biological processes, by regulating cell differentiation, apoptosis, proliferation, and invasion [13, 14]. Lysine-specific histone demethylase 5C (KDM5C) (JARID1C) is a member of this family and regulates gene expression through reducing H3K4 trimethylation activity. It has been firmly established that *KDM5C* has dual roles as both a tumor suppressor and an oncogene. It is a tumor suppressor gene in some cancers, such as renal cancer, breast cancer, cervical cancer, and intrahepatic cholangiocarcinoma [15–17], while also acting as an oncogene in hepatocellular carcinoma and prostate cancer [18, 19]. Moreover, KDM5C is necessary for ovarian development [20]. Recently, Gabory *et al*. also reported that KDM5C is strongly expressed in female placentas but not male placentas [21]. However, studies on the role of KDM5C in the maternal-fetal interface have not yet been published.

In this study, we’ve shown that RM samples have a substantially higher amount of KDM5C in trophoblasts when compared with normal controls. KDM5C overexpression reduces trophoblastic invasion in both in vitro and ex vivo experimental assays. Mechanism-focused research has revealed that KDM5C is an important modulator of TGFβ2 and RAGE expression via demethylation of H3K4me3 at the promoters of the *TGFβ2* and *RAGE* gene. Thus, these findings highlight a crucial role for KDM5C in the pathogenesis of RM disease and establish a relationship between KDM5C and TGFβ2/RAGE in the epigenetic regulation of trophoblasts.

## Results

### KDM5C expression was upregulated in trophoblasts from recurrent miscarriage (RM) patients

**Fig. 1:**
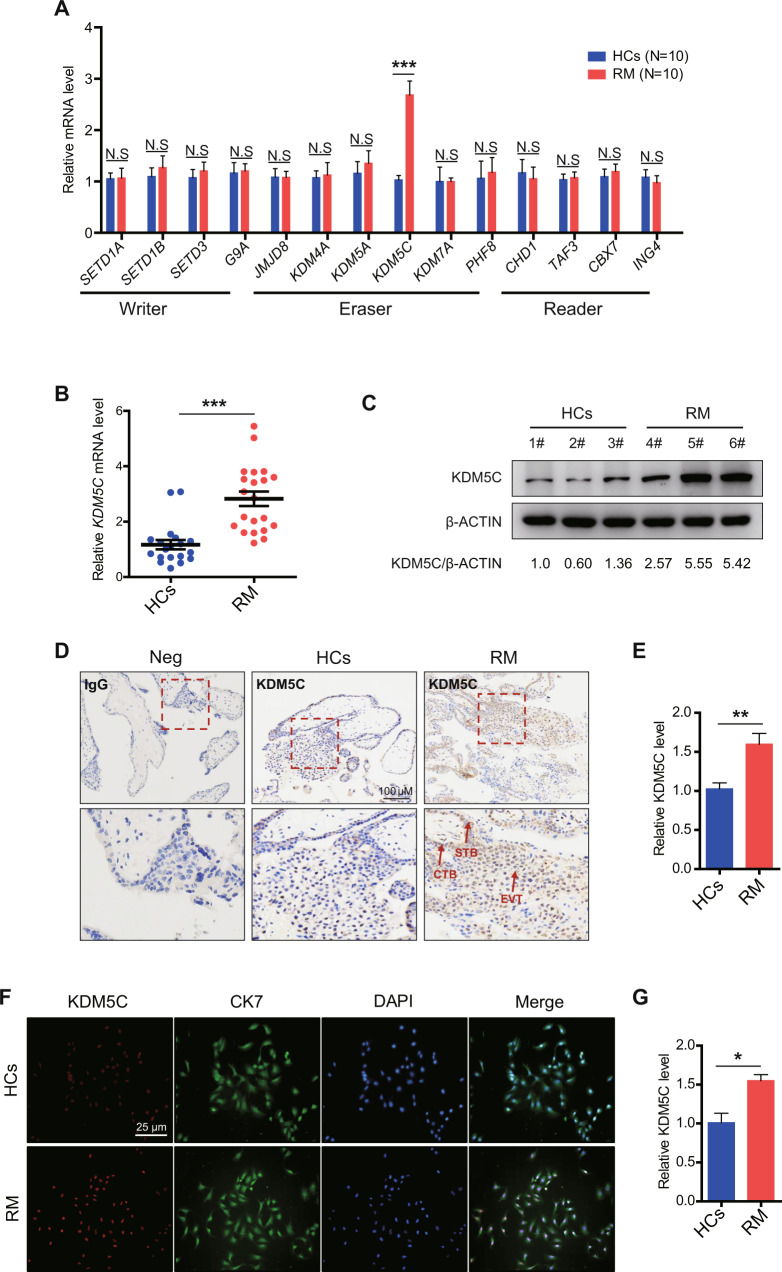
KDM5C is upregulated in placental trophoblasts from RM patients. **A** qRT-PCR assay was performed to detect the levels of mRNA of histone ‘writer’, ‘eraser’, and ‘reader’ related genes in human villi tissues isolated from first trimester RM patients (*n* = 10) or HCs (*n* = 10). **B** Level of *KDM5C* mRNA was determined in villi tissues of RM patients (*n* = 19) and HCs (*n* = 21) using qRT-PCR assay. **C** Western blotting assay of the protein level of KDM5C in HCs (*n* = 3) and RM patient (*n* = 3) villous tissues. **D**, **E** Immunolabeling of maternal villi (cytotrophoblasts, syncytiotrophoblast, and extravillous trophoblasts) with anti-IgG (rabbit) or anti-KDM5C antibody, which was detected using a horseradish peroxidase (HRP) kit. Sections received hematoxylin counter staining, and positive cells were quantified using Image-Pro Plus 6.0 software; Scale bar = 100 μM; *n* = 10 for each group. STB syncytiotrophoblast; CTB cytotrophoblast; EVT extravillous trophoblast. **F** Representative images of KDM5C expression in primary trophoblasts isolated from HCs and RM patients. Positive KDM5C staining was red; CK7 staining, green; and DAPI-stained nuclei, blue. Scale bar = 25 μM. **G** KDM5C expression was measured in primary trophoblasts from HCs group and RM patients group using Lecia confocal SP8 software; *n* = 6 for each group. **A** the ANOVA test; **B**, **E**, **G** Student’s *t* test. Data are shown as the mean ± SEM; *p < 0.05, **p < 0.01, ***p < 0.001.

It has been previously shown that there is a connection between insufficient proliferation and invasion of trophoblasts and the occurrence of early or late RM [7, 22]. To further investigate the role of histone methylation in the pathogenesis of RM, we performed qRT-PCR on villi tissues from recurrent miscarriage (RM) patients (*n* = 10) and healthy controls (HCs, *n* = 10) to assess the expression profiles of histone ‘writer’, ‘eraser’ and ‘reader’ related genes. Our qRT-PCR data found that expression of *KDM5C* mRNA was dramatically higher in RM patients than that from the HCs (Fig. 1A). We further enlarged the samples size of RM patients (*n* = 21) and HCs (*n* = 19), and obtained similar results (Fig. 1B). Interestingly, the expression of *KDM5C* was relatively higher in female placentas than male placentas (Supplementary Fig. 1A), which was consistent with the observation in mouse [21]. Consistently, western blotting assay showed that KDM5C expression at the protein level was upregulated in the villi tissues of RM patient group when compared with samples from HCs (Fig. 1C). To further determine the localization of KDM5C in chorionic villous tissue, immunohistochemical (IHC) analysis was performed, utilizing paraffin-embedding to prepare samples for microscopy. We found that KDM5C is mainly expressed within the nuclei of CTBs and EVTs, the immunohistochemical signal for KDM5C appeared to be stronger in chorionic villus of the RM patients than those of HCs (Fig. 1D, E). Furthermore, expression of KDM5C was higher in RM group compared with the HCs through double immunofluorescence staining of primary cytotrophoblasts, with CK-7 as a marker for trophoblasts (Fig. 1F, G). In conclusion, these data show that the expression of KDM5C is significantly upregulated in trophoblast cells from RM patients.

### KDM5C overexpression decreased trophoblast proliferation and invasion

**Fig. 2:**
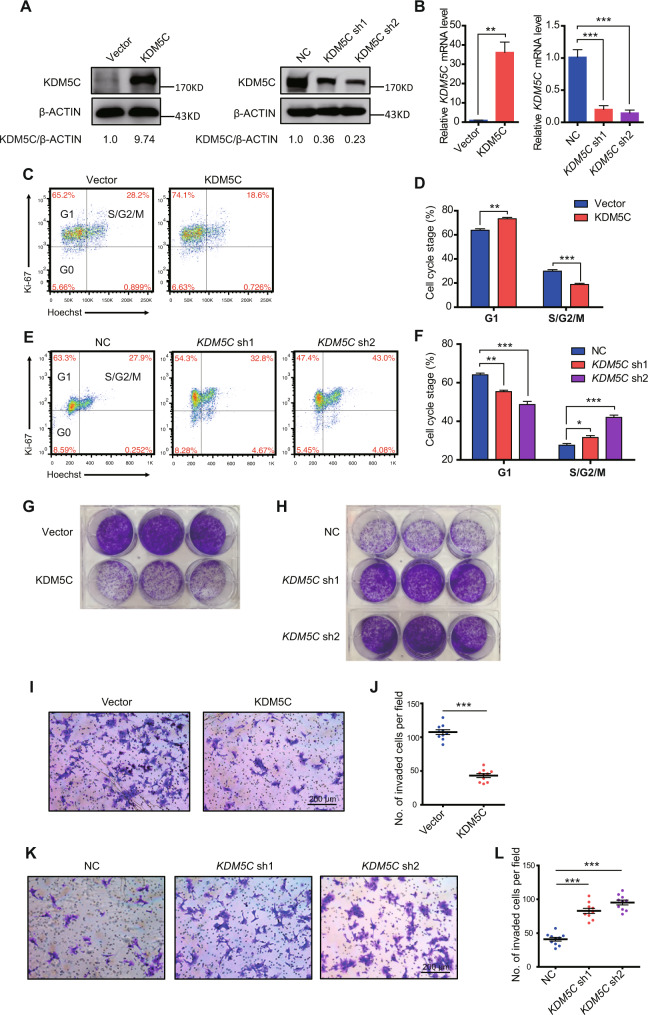
KDM5C decreases trophoblast proliferation and invasion. **A** Western blotting experiments of the protein level of KDM5C in HTR-8 cells stably transduced with vector control, KDM5C-expressing vector, control shRNA, or two *KDM5C* shRNAs. **B** qRT-PCR experiments of *KDM5C* mRNA levels found in HTR-8 cells stably transduced with vector control, KDM5C-expressing vector, control shRNA or two *KDM5C* shRNAs; *n* = 3 for each group. Flow cytometry analyses of the cell cycle status in HTR-8 cells under KDM5C overexpression conditions (**C**, **D**) or in HTR-8 cells under *KDM5C* knockdown conditions (**E**, **F**); **D**, **F**, *n* = 3 for each group. Colony formation assay of HTR-8 cells under KDM5C overexpression conditions (**G**), or of HTR-8 cells upon knockdown of *KDM5C* (**H**). Cell invasion assay of HTR-8 cells under KDM5C overexpression conditions (**I**, **J**) or of HTR-8 cells upon knockdown of *KDM5C* (**K**, **L**); scale bar = 200 μM; **J**, **L**, *n* = 10 for each group. **B**, left panel, Student’s *t* test; **B**, right panel, the ANOVA test; **D**, **F**, **L**, the ANOVA test; **J**, Student’s *t* test. Data are shown as the mean ± SEM; *p < 0.05, **p < 0.01, ***p < 0.001.

To further determine KDM5C activity in human trophoblasts, HTR-8/SVneo (HTR-8) cell line transfected with a KDM5C-expression cassette or two different *KDM5C* shRNAs to achieve stable overexpression or knockdown, respectively (Fig. 2A, B). Flow cytometry analyses showed that overexpression of KDM5C strongly inhibited the cell cycle, whereas knockdown of *KDM5C* accelerated the cell cycle of HTR-8 cells (Fig. 2C–F). Specifically, cell cycle analysis showed that KDM5C overexpression resulted in a significant increase in the percentage of cells in G1 phase and a significant decrease in the percentage of cells in G2/M phase, which indicated that upregulation of KDM5C induced cell cycle arrest at G2/M phase (Supplementary Fig. 2A, B). In contrast, the cell portion in G2/M phase was increased after the downregulation of KDM5C (Supplementary Fig. 2C, D). However, KDM5C overexpression or knockdown did not exert an apparent effect on apoptosis of HTR-8 cells (Supplementary Fig. 2E–H). Interestingly, overexpression of KDM5C resulted in decreased colony-forming potential, whereas *KDM5C* knockdown appeared to enhance the colony-forming potential of HTR-8 cells (Fig. 2G, H). The cell counting kit-8 (CCK8) assay further confirmed that cell proliferation was decreased by KDM5C overexpression and enhanced by *KDM5C* knockdown (Supplementary Fig. 2I–J). In addition, Matrigel invasion assays show that overexpression or knockdown of KDM5C dramatically weakened or improved the invasiveness of HTR-8 cells (Fig. 2I–L).

To further investigate the role of KDM5C in trophoblast cells, we used the neoplastic trophoblast cell line, JAR, which is similar to early human trophoblast cells [23]. Consistent with the observation in HTR-8 cells, KDM5C overexpression decreased the colony-forming capability, whereas KDM5C knockdown increased the colony-forming capability of JAR cells (Supplementary Fig. 3A–D). Moreover, the colony-forming capability of JAR cells was decreased by *KDM5C* overexpression, whereas increased by *KDM5C* knockdown (Supplementary Fig. 3E, F). These findings support that KDM5C is important in the repression of trophoblastic proliferation and invasion.

### KDM5C overexpression reduces the invasive ability of EVTs in an ex vivo model

**Fig. 3:**
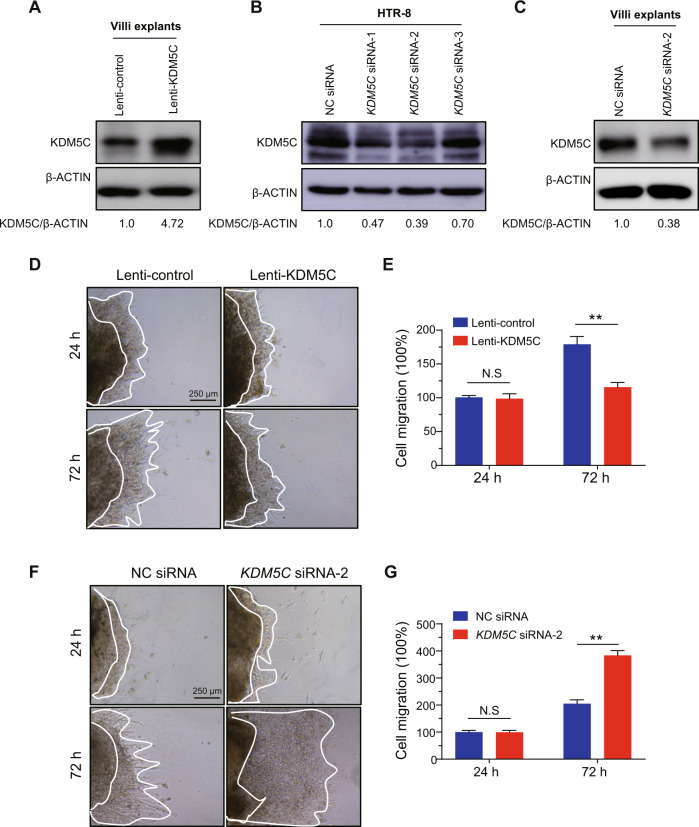
KDM5C inhibits trophoblast outgrowth in a villi explants model. **A** The villi explants were seeded in Matrigel-coated plates, and western blotting assay was performed on KDM5C protein from explants treated with either lenti-control or lenti-KDM5C for 72 h. **B** Level of KDM5C protein was determined in HTR-8 cells transduced with control or *KDM5C* siRNAs using western blotting assay. **C** The villi explants were plated on Matrigel-coated plates, and western blotting assay was performed on KDM5C protein in extravillous explants transduced with either NC siRNA or *KDM5C* siRNA-2 for 72 h. **D**, **E** Villi explants from HCs were cultured on Matrigel-coated plates. Time course images of the villi explants treated with either lenti-control or lentiviral vector carrying the *KDM5C* gene (lenti-KDM5C) were taken using light microscopy after 24 and 72 h of culture ex vivo; Scale bar = 250 μM. Statistical assay of villous tip migration (%), *n* = 6 for each group. **F**, **G** Villi explants were cultured in Matrigel-coated plates. Time course images of the explants transfected with either control siRNA or *KDM5C* siRNA-2 were obtained using microscope after 24 and 72 h of culture ex vivo; Scale bar = 250 μM. Statistical assay of villous tip migration (%), *n* = 6 for each group. Data are shown as the mean ± SEM; Student’s *t* test was used to evaluate the statistical significance; **p < 0.01.

To better understand KDM5C activity within EVTs ex vivo, we utilized villi explants collected from the first-trimester placenta villi (at a gestation window of 6–10 weeks) that were plated on Matrigel-coated culture dishes. The explants were then treated with lenti-control, a lentiviral vector expressing the *KDM5C* gene (lenti-KDM5C), negative control small interfering RNA (NC siRNA) or *KDM5C* siRNA, respectively. The overexpression efficiency of KDM5C in placental villi was confirmed by western blotting assay at 72 h of ex vivo culture (Fig. 3A). To further study *KDM5C* knockdown efficacy, HTR-8 cells were transfected with *KDM5C* siRNA 1–3 oligonucleotides, and we found that KDM5C expression was decreased by *KDM5C* siRNA-1 and siRNA-2 transfection, and *KDM5C* siRNA-2 decreased the expression of KDM5C more significantly than siRNA-1 (Fig. 3B). Therefore, the explants were further treated with control siRNA or *KDM5C* siRNA-2. The KDM5C knockdown efficacy in placental villi was confirmed by western blotting assay at 72 h of ex vivo culture (Fig. 3C). Next, we investigated whether KDM5C expression has any effect on trophoblast migration ex vivo. Explants obtained from first-trimester placentas (at a gestation window of 6–10 weeks) were anchored into Matrigel-coated plates, and after 24 h, the explants began to display outgrowth. At the 24 h time point, we found there were no significant differences between the control group and the lenti-KDM5C group. By 72 h of ex vivo culture, KDM5C overexpression had dramatically inhibited the migratory ability of the explants (Fig. 3D, E). By contrast, *KDM5C* knockdown had significantly extended the migration distance of the explants (Fig. 3F, G). These data indicate that expression of KDM5C inhibits trophoblast outgrowth, and further suggests that KDM5C plays a critical role in early placental development.

### KDM5C overexpression inhibits substantial regulators of trophoblast proliferation and invasion

**Fig. 4:**
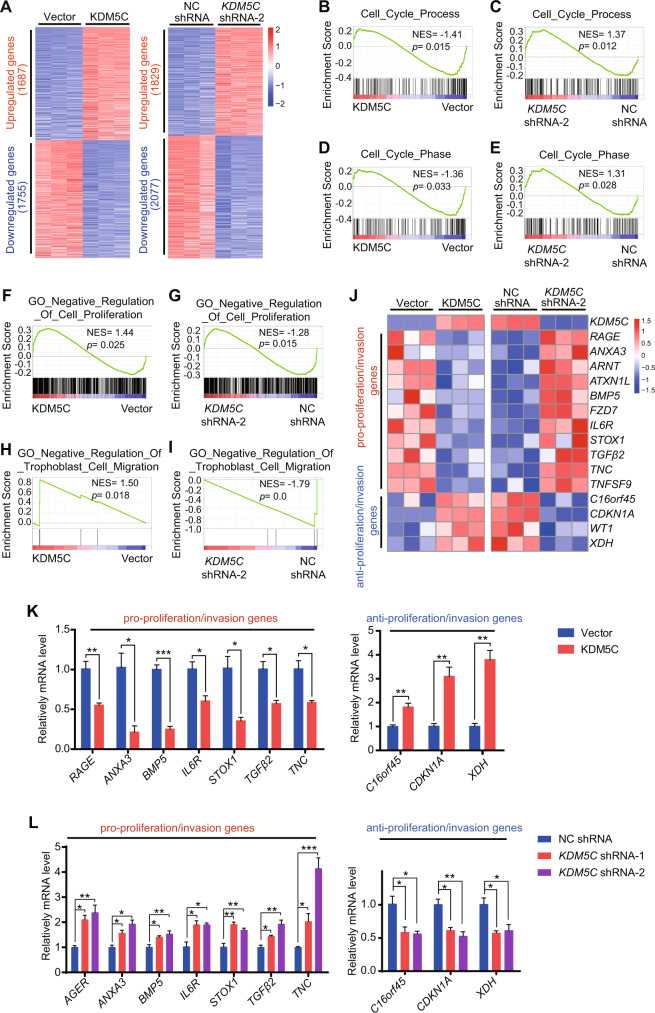
KDM5C inhibits substantial regulators of trophoblast proliferation and invasion. **A** Heatmap indicating differentially expressed genes between HTR-8 cells stably transduced treated with either control vector or KDM5C overexpression vector, or between HTR-8 cells treated with either NC shRNA or *KDM5C* shRNA-2. GSEA of the expression profile of HTR-8 cells under KDM5C overexpression or knockdown conditions using a cell cycle process-associated signature (**B**, **C**), cell cycle phase-associated signature (**D**, **E**), cell proliferation-negative associated signature (**F**, **G**), and trophoblast cell migration-associated signature (**H**, **I**). **J** Heatmap indicating differential gene expression of HTR-8 cells under KDM5C overexpression (left panel) or knockdown (right panel) conditions, which focused on a set of pro-proliferation/invasion genes and anti-proliferation/invasion genes. qRT-PCR assay of the mRNA levels of genes in HTR-8 cells stably transduced with vector control, KDM5C-expressing vector (**K**), control shRNA or two *KDM5C* shRNAs (**L**); *n* = 3 for each group. **K** Student’s *t* test; **L** the ANOVA test. Data are shown as the mean ± SEM; *p < 0.05, **p < 0.01, ***p < 0.001.

To elucidate the regulatory functions of KDM5C in trophoblasts, we performed RNA-sequencing analysis of HTR-8 cells that had been stably transduced with KDM5C-expression cassette or with *KDM5C* shRNA (Fig. 2A, B), and compared them with empty vector or NC shRNA, respectively. Our analysis identified ~3000–4000 differentially expressed genes in each condition (Fig. 4A and Supplementary Tables 1, 2). Differentially expressed genes were then assessed using gene ontology (GO) based on the Database for Annotation, Visualization, and Integrated Discovery (DAVID) database. Consistent with the functional studies above, GO analysis found that genes that were either upregulated upon KDM5C overexpression or downregulated upon *KDM5C* knockdown were revealed to be enriched within functional categories associated with negative regulation of cell cycle or trophoblast migration. In contrast, the genes downregulated upon KDM5C overexpression or genes upregulated upon *KDM5C* knockdown were revealed to be enriched within functional categories associated with positive regulation of the cell cycle and trophoblast migration (Supplementary Fig. 4A). Next, we developed a gene set enrichment analysis (GSEA) to identify molecular pathways that could have been perturbed by a gain or loss of function of associated with altered KDM5C expression. GSEA found that gene signatures related to cell cycle (Fig. 4B, D), cell proliferation (Fig. 4F), and trophoblast migration (Fig. 4H) in KDM5C-overexpressed HTR-8 cells showed significant negative enrichment. Conversely, GSEA showed that gene signatures related with cell cycle (Fig. 4C, E), cell proliferation (Fig. 4G), and trophoblast migration (Fig. 4I) after *KDM5C* knockdown were positively enriched. Nevertheless, the signatures of chorionic trophoblast cell differentiation were not altered by KDM5C overexpression or knockdown (Supplementary Fig. 4B, C).

As expected, overexpression of KDM5C or knockdown of *KDM5C* in HTR-8 cells resulted in altered mRNA levels of essential regulators of cell proliferation and invasion, respectively, which was verified by qPCR assays. The overlap of these two subsets identified remarkably sensitive responsive genes. The proliferation and invasion genes, including *ARNT* [24], *BMP5* [25], *FZD7* [26], *IL6R* [27], *STOX1* [28], and *TNC* [29], were downregulated under KDM5C overexpression conditions and upregulated under *KDM5C* knockdown conditions (Fig. 4J–L). In contrast, proliferation and invasion blockers, *CDKN1A* [30] and WT1 [31], were upregulated when KDM5C was overexpressed and downregulated when *KDM5C* was knocked down (Fig. 4J–L). WT1 was first identified as a tumor suppressor in nephroblastoma, while late studies revealed that it also acts as an oncogene in leukemia [32], indicating that it may regulate cell proliferation and invasion in a cell type-dependent manner. Importantly, two of these genes, *TGFβ2* and *RAGE*, are critical regulators of proliferation and invasion in various cell types [33–36]. KDM5C overexpression leaded to a significant decrease in the both *TGFβ2* and *RAGE* mRNA expression in HTR-8 cells, and *KDM5C* knockdown increased the expression of both *TGFβ2* and *RAGE* mRNA (Fig. 4J–L). Altogether, these results suggest that KDM5C expression regulates a subset of genes involved in trophoblast proliferation and invasion.

### KDM5C overexpression regulates dynamic H3K4me3 modification in the promoters of target genes

**Fig. 5:**
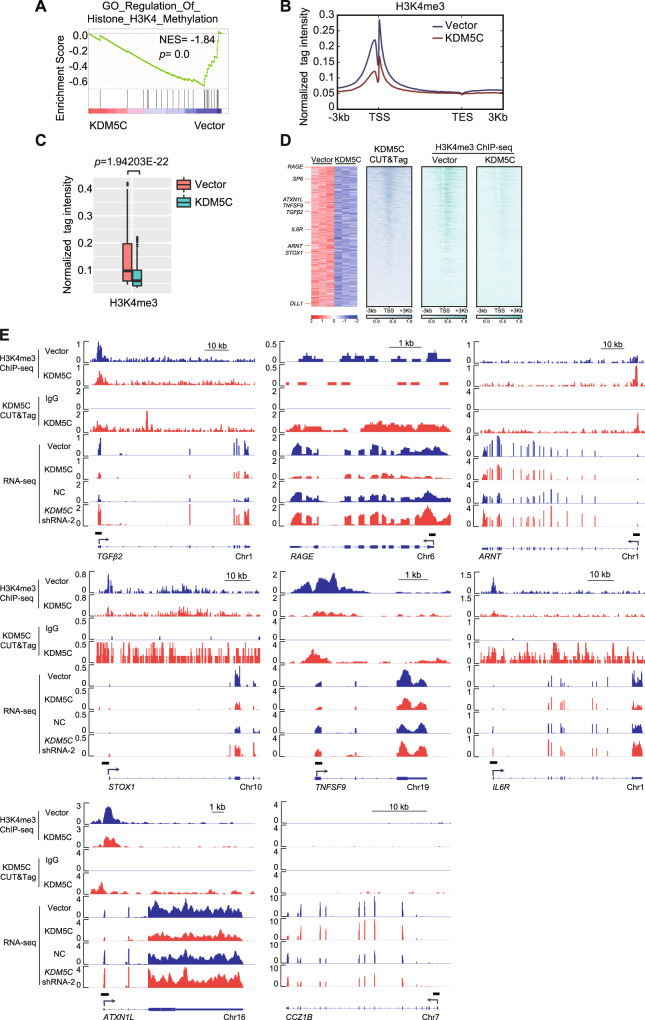
KDM5C regulates dynamic H3K4me3 in the promoters of target genes. **A** GSEA of the expression profile of HTR-8 cells that were stably transduced with either control vector or KDM5C overexpression vector via a histone H3K4 methylation-associated signature. **B** Pattern of H3K4me3 signal profile within ±3 kb genomic regions from anchors on the transcriptional start site (TSS) and transcriptional termination site (TTS) of HTR-8 cells stably transduced with either control vector or KDM5C overexpression vector. **C** Box plots indicating the changes in H3K4me3 occupancy around promoters (within 3 kb genomic regions flanking the TSS) of HTR-8 cells stably transduced with either control vector or KDM5C overexpression vector. **D** Heatmap representation of the downregulated genes in HTR-8 cells under KDM5C overexpression conditions. The binding profiles of KDM5C or H3K4me3 within 3 kb genomic regions flanking the TSS of each gene are also shown. **E** H3K4me3 ChIP-seq in HTR-8 cells stably transduced with either control vector or KDM5C overexpression vector, IgG or KDM5C CUT&Tag in HTR-8 cells stably transduced with KDM5C overexpression vector, and RNA-seq of HTR-8 cells stably transduced with either control vector or KDM5C overexpression vector. Genome browser tracks representing sites for H3K4me3 binding or KDM5C at *TGFβ2*, *RAGE*, *ARNT*, *STOX1*, *TNFSF9*, *IL6R*, *ATXN1L,* or *CCZ1B* gene loci.

Due to KDM5C being a histone H3K4-specific demethylase, we expected that the level of H3K4me3’s direct target genes would decrease at regulatory regions following KDM5C overexpression. GSEA supported this notion by detecting a negative enrichment in gene signatures related with the regulation of H3K4 methylation in KDM5C-overexpressed HTR-8 cells (Fig. 5A). Furthermore, we performed ChIP-seq assay to identify any possible alterations in H3K4me3 after KDM5C overexpression in HTR-8 cells. Analysis of the average H3K4me3 levels of genes that were differentially methylated revealed that H3K4me3 methylation levels were significantly decreased in both promoters and gene bodies, especially in promoter regions, upon KDM5C overexpression (Fig. 5B, C and Supplementary Fig. 5A). We conducted a genome-wide study of H3K4me3 localization and found that the KDM5C-downregulated genes experienced a loss of H3K4me3 upon KDM5C overexpression and were marked by the presence of KDM5C within 3 kb upstream or downstream of transcription start sites (TSSs) within promoters (Fig. 5D). As revealed by the genomic browser track of ChIP-seq data, which was confirmed by ChIP-qPCR analyses, the decrease in H3K4me3 was clear near the promoters of the representative downregulated regulators of trophoblast proliferation and invasion, such as *TGFβ2*, *RAGE, ARNT, STOX1*, *TNFSF9, IL6R* and *ATXN1L, and* but not in *CCZ1B*’s gene locus, which we used as a negative control (Fig. 5E and Supplementary Fig. 5B). In parallel, KDM5C CUT&Tag and ChIP-qPCR analysis of possible sites for KDM5C-binding, where H3K4me3 accumulations were significantly decreased in KDM5C-overexpressed HTR-8 cells, showed that regulators of trophoblast proliferation and invasion were genes directly targeted by KDM5C in HTR-8 cells (Fig. 5E and Supplementary Fig. 5D). Expectedly, *KDM5C* knockdown increased H3K4me3 but decreased KDM5C abundance around these potential KDM5C-occupying sites (Supplementary Fig. 5C, E). These data indicates that KDM5C regulates genes that play a crucial role in proliferation and invasion of trophoblasts, primarily through altering the histone methylation status of H3K4me3 in the promoters of target genes.

### TGFβ2 and RAGE represent two major targets of KDM5C that repress trophoblast proliferation and invasion

**Fig. 6:**
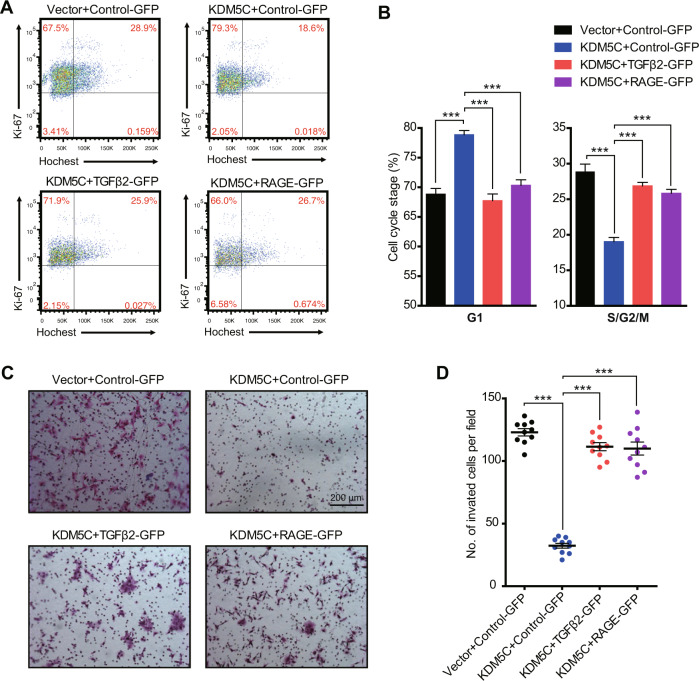
TGFβ2 and RAGE represent two major targets of KDM5C to repress trophoblast proliferation and invasion. HTR-8 cells stably transfected with either control vector or KDM5C overexpression vector were additionally lentivirally transduced with control-GFP, TGFβ2-GFP, or RAGE-GFP, and the cell cycle status was then analyzed using flow cytometry (**A**, **B**), and the invasive ability of HTR-8 cells was determined using Image-Pro Plus 6.0 software (**C**, **D**); scale bar = 200 μM; B, *n* = 3 for each group; D, *n* = 10 for each group. The ANOVA test was used to evaluate the statistical significance; ***p < 0.001.

Our findings revealed that *TGFβ2* and *RAGE* are direct downstream target genes of KDM5C, raising the possibility that KDM5C might regulate trophoblast proliferation and invasion through the regulation of TGFβ2 or RAGE expression. Flow cytometry analyses found that overexpression of TGFβ2 or RAGE reversed cell cycle arrest that had been initiated by KDM5C overexpression in HTR-8 cells (Fig. 6A, B and Supplementary Fig. 6A, B). Moreover, overexpression of TGFβ2 or RAGE also reversed the decrease in trophoblast invasion seen under KDM5C overexpression conditions (Fig. 6C, D). Thus, our results support that KDM5C negatively regulates TGFβ2 and RAGE to mediate trophoblast proliferation and invasion.

### TGFβ2 and RAGE negatively correlate with KDM5C in RM disease

**Fig. 7:**
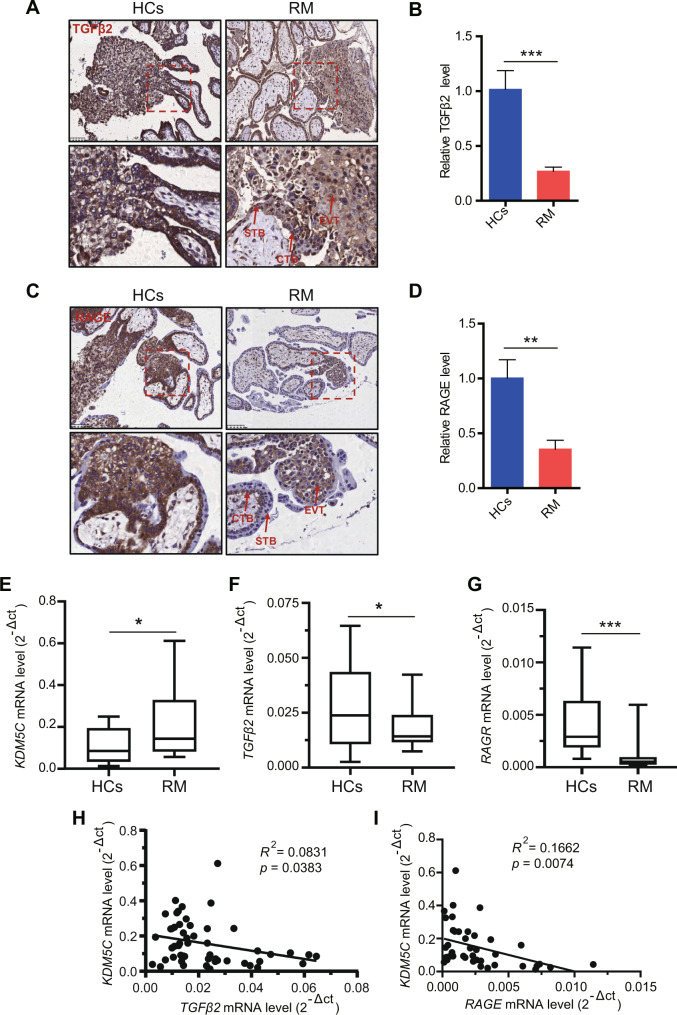
TGFβ2 and RAGE negatively correlate with KDM5C in RM patients. Immunolabeling of villi tissue from HCs and RM patients with anti-TGFβ2 antibody (**A**, **B**), or anti-RAGE antibody (**C**, **D**), which were visualized using a HRP kit. Sections were hematoxylin counterstained, and cell positivity was quantified using Image-Pro Plus 6.0 software (*n* = 10); Scale bar = 100 μM. STB, syncytiotrophoblast; CTB, cytotrophoblast; EVT: extravillous trophoblast. The mRNA levels of *KDM5C* (**E**), *TGFβ2* (**F**), and *RAGE* (**G**) found in villi from HCs (*n* = 25) and RM patients (*n* = 29) were detected using qRT-PCR. (H, I) level of *KDM5C* mRNA was negatively associated with level of *TGFβ2* or *RAGE* mRNA in RM patients’ villous tissues and HCs. Data are shown as the mean ± SEM; Student’s *t* test was used to evaluate the statistical significance; *p < 0.05, **p < 0.01, ***p < 0.001.

We further surveyed expression levels of TGFβ2 and RAGE in villous tissues. IHC staining showed that TGFβ2 was expressed in CTBs, STB, and EVTs (Fig. 7A), and RAGE was highly localized to the CTBs and EVTs (Fig. 7C). Both TGFβ2 and RAGE had significantly lower expression in the RM group compared with the control group (Fig. 7A–D). Furthermore, level of *KDM5C*, *TGFβ2*, and *RAGE* mRNA expression in HCs and RM patients were determined using qRT-PCR, and these findings showed that *KDM5C* mRNA level was elevated in RM patients when compared with HCs (Fig. 7E). By contrast, both *TGFβ2* and *RAGE* mRNA levels were downregulated in RM patients (Fig. 7F, G). Correlation analysis revealed a negative correlation between *KDM5C* mRNA level and *TGFβ* or *RAGE* mRNA level within villi tissues from RM patients (Fig. 7H, I). These results suggest that KDM5C is negatively related with TGFβ2 or RAGE levels in RM patients, suggesting a way in which KDM5C might contribute to RM pathogenesis.

### Overexpression of KDM5C in mouse placenta contributes to the embryo resorption

**Fig. 8:**
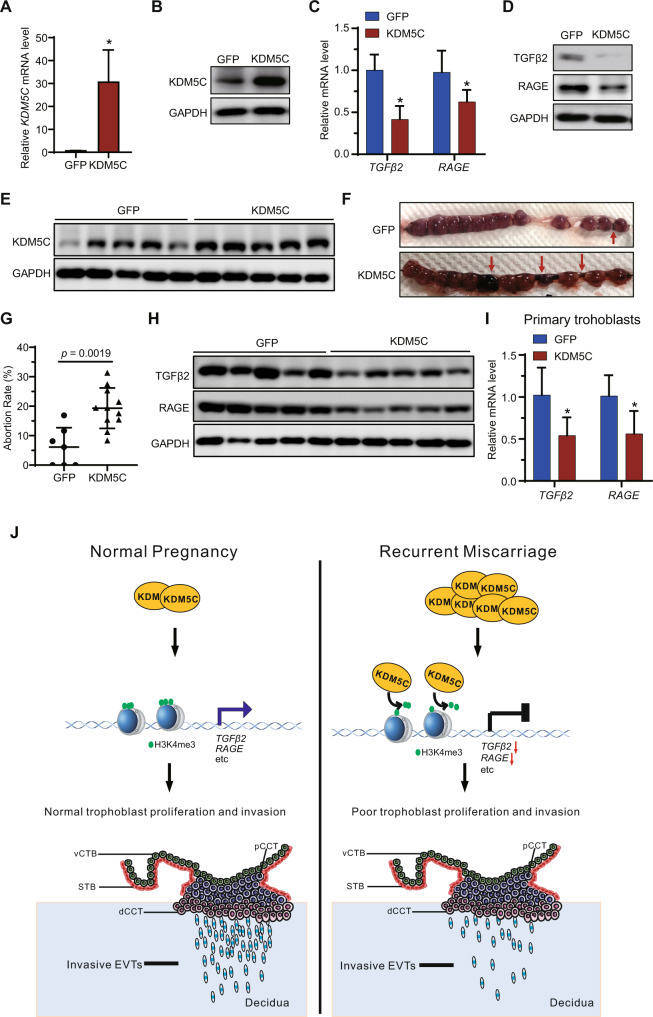
KDM5C overexpression promotes embryo resorption in a mouse model. Mouse trophoblast stem cells were transduced with adenovirus-mediated GFP (Ad-GFP) or adenovirus-mediated mouse KDM5C gene (Ad-KDM5C). Level of KDM5C (**A**, **B**), TGFβ2 and RAGE. (**C**, **D**) expression in trophoblast stem cells was detected through qRT-PCR assay at the mRNA level and western blotting at the protein level; C, *n* = 3 for each group. **E** Western blotting to determine the level of KDM5C expression in mouse placenta with Ad-GFP or Ad-KDM5C treatments. **F**, **G** Embryo-resorption rates in mice treated with Ad-GFP (*n* = 7) or Ad-KDM5C treatments (*n* = 11). Representative macroscopic imaging of uteri at gestational age E 11.5 in pregnancies that received various treatments. **H** Protein level of TGFβ2 and RAGE expression in mouse placenta with Ad-GFP or Ad-KDM5C treatments as determined by western blotting. **I** Level of *TGFβ2* and *RAGE* mRNA detected in primary trophoblasts isolated from Ad-GFP- and Ad-KDM5C-injected mice were determined by qRT-PCR; *n* = 5 for each group. **J** In normal pregnancies, decreased expression of KDM5C is associated with higher H3K4me3 methylation in the promoters of target genes, such as *TGFβ2* and *RAGE*, therefore promoting transcription of the *TGFβ2* and *RAGE* gene, which keeps trophoblasts in a normal state of proliferation and invasion (Left). In RM, this signal transduction pathway is altered due to an increase in the expression of KDM5C, which binds to the promoters of certain target genes, such as *TGFβ2* and *RAGE*, and reduces the local H3K4me3 constitutively to repress transcription of these target genes, ultimately resulting in inhibition of trophoblast invasion and proliferation and the development of RM (Right). dCCT distal cell column trophoblast; pCCT proximal cell column trophoblast; STB syncytiotrophoblast; vCTB villous cytotrophoblast; EVTs extravillous trophoblasts. Data are shown as the mean ± SEM; Student’s *t* test was used to evaluate the statistical significance; *p < 0.05.

To further examine whether TGFβ2 or RAGE are downstream genes of KDM5C in mouse trophoblasts, mouse trophoblast stem cells were treated with the adenovirus-mediated mouse KDM5C (Ad-KDM5C) or adenovirus-mediated GFP (Ad-GFP) for 72 h (Fig. 8A, B). Expression of TGFβ2 and RAGE was found to be decreased under KDM5C overexpression conditions (Fig. 8C, D). To further determine whether upregulation of KDM5C in mouse placenta could induce abortion, the mouse was injected Ad-GFP or Ad-KDM5C via the tail vein at embryonic day (E) 3.5. At embryonic day (E) 11.5, the level of KDM5C expression in the mouse placenta was detected using western blotting (Fig. 8E). Our results found that KDM5C overexpression in the mouse placenta promoted the embryo-resorption at embryonic day (E) 11.5 (Fig. 8F, G). Furthermore, the expression of TGFβ2 and RAGE in murine placentas transfected with Ad-GFP or Ad-KDM5C was detected using western blotting. The results showed that expression of TGFβ2 and RAGE was decreased in mouse placenta with Ad-KDM5C transfection (Fig. 8H). We verified our findings by qRT-PCR analysis, which showed that expression of *TGFβ2* and *RAGE* was decreased in the mouse primary trophoblasts treated with Ad-KDM5C transfection compared to Ad-GFP transfection (Fig. 8I). Together, these results indicate that KDM5C overexpression in mouse placenta promotes embryonic resorption.

## Discussion

Recurrent miscarriage (RM) is frustrating for the physicians and heartbreaking for the patients, which brings physical and psychological suffering to the mothers and families impacted [37]. Although plenty of clinical efforts have been made towards increase the rate of live births amongst women struggling with RM, no therapeutic strategies have been proven effective [38]. Therefore, it is urgent that scientific researchers continue work to identify the underlying molecular mechanisms promoting RM. Our study revealed the biological function of KDM5C in promoting proliferative and invasive ability of trophoblast, suggesting a potential role for KDM5C in the pathogenesis of RM (Fig. 8J).

Study have found that, in the first trimester, EVTs have a high invasive potential, invading the uterine epithelium and spiral arteries to establish the maternal-fetal interface. Insufficient EVT invasion can increase the risk of recurrent miscarriages (RM). Therefore, to investigate the pathogenesis of RM, it is important to compare the differences of varying gene patterns in placenta with or without RM disease. Previous studies have found that impaired EVT invasion at early pregnancy contributes to the pathogenesis of RM [39–41]. Recently, epigenetic modifications have been linked to factors already associated trophoblast function. Yu et al. have reported that H3K36me3/2 demethylase KDM4C contributes to trophoblast-like stem cell formation via activating CDX2 expression [42]. Moreover, Meister et al. found a reduced co-expression with H3K4me3 and H3K9ac in the EVT cells in preeclampsia (PE) [43]. However, to date, little is known about the role of epigenetic modifications in EVT invasion under the context of RM. In gastric cancer cells, overexpression of KDM5C inhibited the expression of p53, and thereby enhanced tumor formation [44]. While, Zhang et al. recently reported that KDM5C was downregulated in intrahepatic cholangiocarcinoma cells (ICC), and its expression and function were required for inhibition of ICC proliferation and invasion and tumor repression through negatively regulating the expression of FASN [45]. By contrast, in this study, we first found that lysine-specific demethylase 5C (KDM5C) level was higher in the placental tissue from RM patients than that of age- and gestational-matched HCs, suggesting that KDM5C is disrupted under pathological conditions. Moreover, multiple lines of evidence, including in vitro cell migration and invasion assays, ex vivo extravillous explant culture experiments, and analyses of human RM specimens, support the role of KDM5C in mediating trophoblast via regulating TGFβ2 and RAGE expression, which contrasts with its previously described role as an oncogene in studies on hepatocellular carcinoma, prostate cancer, and acute myeloid leukemia. These observations suggest KDM5C has other regulatory effects, including on cell survival, proliferation and invasion. However, the more detailed molecular mechanism of KDM5C in regulation trophoblast invasion warrants further investigation.

The development of normal maternal-fetal circulation and, thus, a healthy gestation in humans, depends on the trophoblast invasion into the endometrial and inner-third of the myometrium during early pregnancy [1]. During RM, however, these trophoblasts possess abnormal proliferative and invasive properties that contribute to pathogenesis [6, 7]. Recently, TGFβ2 and RAGE were shown to facilitate trophoblast proliferation and invasion. TGFβ2, but not TGFβ1, was found to be highly expressed in EVTs; therefore, TGFβ2 may act as a marker for EVTs in vitro and in situ. TGFβ2 expression was detected to be lower in trophoblastic and decidual cells from miscarriages when compared to uncomplicated pregnancies [46]. A recent study showed that TGFβ2, which was a downstream target of miR-193b-3p, can enhance HTR-8 cell migration and invasion [30]. RAGE may also be used as a biomarker to predict and diagnose RM as it was showed to be significantly downregulated in serum RM patients compared with HCs [47]. RAGE has been found to be elevated in various malignancies and is generally related to an unfavorable outcome, and blockade of RAGE has been shown to suppress cancer cell proliferation, invasion, and metastases [35, 36]. Here, we revealed that overexpression KDM5C inhibited trophoblast proliferation and invasion by reducing H3K4me3 modification of the promoters for target genes, including *TGFβ2* and *RAGE*, demonstrating that KDM5C may be a driving factor in the pathogenesis of RM through the inhibition of TGFβ2 and RAGE expression.

In this study, we supply new insight on RM pathogenesis by exploring the function of KDM5C, which was shown to regulate the proliferation and invasion of trophoblasts during early gestation, primarily through alterations in the histone methylation status of H3K4me3 in the promoters of target genes. Among those genes, we identified TGFβ2 and RAGE to be strictly regulated by KDM5C in vitro and ex vivo. KDM5C, by its regulating of TGFβ2 and RAGE expression, might play a critical role in the RM disease, suggesting that therapeutically targeting KDM5C may have potential as a future treatment for RM.

## Material and methods

### Patient information

This study includes 31 recurrent miscarriage (RM) patients from the Obstetrics and Gynecology Hospital of Fudan University between December 2018 and July 2019. Conditions under which the patients were excluded include: (1) karyotype analyses of the abortuses or parents were proved to be abnormal; (2) pelvic examination and ultrasound found the presence of cervical incompetence or uterine abnormalities; (3) no symptoms of endocrine or metabolic diseases (e.g., diabetes, hyperthyroidism, and hypothyroidism); (4) hyperandrogenaemia, hyperprolactinaemia and luteal phase defects were ruled out by comprehensive hormonal status assessment. Healthy controls (HCs) included 26 women. The gestational weeks were the same as the RM group. The HCs group is no history of pre-eclampsia, preterm labor, or spontaneous abortion, and the karyotype was normal. All abortuses were obtained from healthy controls through artificial abortions. Patient information is available in Supplementary Table 3.

### Cell Culture

The HTR-8/SVneo cell line [48], was a human invasive EVTs line, was graciously provided by Dr. PK Lala (University of Western Ontario, Canada). The cells were cultured in DMEM/F12 with 10% fetal bovine serum (FBS; Gibco). JAR cells were obtained from the cell bank at the Chinese Academy of Sciences (Shanghai, China) with the original source being the American Type Culture Collection (ATCC) (Manassas, VA, USA) and cultured in 1640 complete medium supplemented with 10% FBS plus Penicillin/ Streptomycin antibiotics in 5% CO_2_ at 37 °C. Mouse trophoblast stem cell (a gift from Dr. Shaorong Gao at Tongji University) were cultured in media and conditions previously described [49]. These cells were recently authenticated by STR profiling and tested for mycoplasma contamination.

### Primary trophoblast isolation

Primary trophoblasts were isolated from samples of villi tissue collected at 6–10 weeks’ gestation, as previously described [50]. In brief, the cell suspensions were obtained using a 70 μM filter, and Cytotrophoblasts (CTBs) were purified using an EasySep™ PE Positive Selection Kit II (STEMCELL Technologies) with fluorescent anti-CD49f (PE) antibodies (Miltenyi Biotec). Purified trophoblast cell culture had a purity of 95%, which was determined by flow cytometry for cytokeratin 7-positive, and vimentin-negative cells. The cells were further seeded in the wells of 12-well plates at a concentration of 2 × 10^5^ cells/mL and cultured in DMEM/F12 (GE Healthcare Life Sciences).

### Lentivirus transduction

Plasmids used to package lentivirus, including psPAX2 and pMD2.G, were obtained from Addgene (Cambridge, MA). For KDM5C overexpression, 5 μg pMD2.G, 10 μg psPAX2, and 5 μg empty vector plasmids (pLVX-IRES-Puro) or 12 μg KDM5C constructs (pLVX-IRES-KDM5C-Puro) were co-transfected into a 100 mm cell culture dish containing HEK-293T cells and Sofast Transfection Reagent (Sunmabio, China). After transfection, lentiviral particles were harvested at 48 and 72 h, and concentrated using an XE-90 Super Speed Centrifuger (Beckman). The HTR-8 cell line or primary trophoblast cells were subsequently infected with our concentrated lentiviruses. For TGFβ2 or RAGE overexpression, the HTR-8 cells were cultured with lentiviruses containing GFP control vector (pLVX-IRES-GFP), or TGFβ2 (pLVX-IRES-TGFβ2-GFP), or RAGE (pLVX-IRES-RAGE-GFP). Five days after infection, cells that were GFP^+^ were isolated by FACS.

The lentivirus-mediated RNA interference of *KDM5C* was cloned in pLKO.1 TRC (Addgene). A scrambled siRNA precursor (Scr) was used as a negative control. The sequences of shRNAs used to target *KDM5C* are as follows: *KDM5C* shRNA-1: 5'-TCGCAGAGAAATC-GGGCATTT-3'; *KDM5C* shRNA-2: 5'-AGTACCTGCGGTA- TCGGTATA-3'.

### Adenovirus

Recombinant adenovirus encoding mouse KDM5C (Ad-KDM5C) and adenovirus encoding GFP (Ad-GFP), was purchased from oBiO Technology Corp., Ltd (Shanghai, China), and stored at −80 °C.

### Animal experiments

C57BL/6J mice were obtained from SLRC Laboratory Animal (Shanghai, China) and mice were housed at light/dark cycles of 12 h with relative humidity. Female C57BL/6J mice were mated with male mice at 8 weeks. The presence of a vaginal plug was used the indicate the first day of pregnancy (E 0.5). Pregnant mice were randomized into 2 groups (*n* = 12 mice, respectively): adenovirus-mediated KDM5C or control adenovirus-mediated GFP (Ad-GFP) injected with 1 × 10^9^ plaque forming unit (PFU) via the tail vein on day E 3.5, which is when the early blastocyst should attach to the endometrial epithelium during murine pregnancy. On day E 11.5 mice were sacrificed. Phenotyping in mouse was performed blinded.

### Invasion Assay

Matrigel invasion assay was performed to evaluate the invasive ability of trophoblasts in vitro, as previously described [22]. Briefly, cell culture inserts were coated with 25 µL of Matrigel™ (Corning, New York, USA) and placed in a 24-well plate. HTR-8 cells were stably transfected with lentivirus containing NC shRNA, KDM5C shRNA-1, KDM5C shRNA-2, control vector, or KDM5C overexpression vector for 48 h. Then, 1.2 × 10^5^ cells/200 µL of DMEM/F12 were seeded into the upper chamber of each well. 800 µL of DMEM/F12 plus 15% FBS were filled to lower wells, and then the cells were cultured for 48 h. The upper inserts were washed in ice-cold PBS three times, and the non-invading cells were removed with a cotton bud. The cells on the lower surface of the inserts were fixed with 4% paraformaldehyde (PFA) and stained with crystal violet. The images were taken using an inverted phase-contrast microscope (Leica). The experiments were independently repeated three times.

### Quantitative real-time PCR (qRT-PCR) and RNA-Sequencing

Total cellular RNA was extracted using the RNeasy mini kit (QIAGEN) following the manufacturer’s instruction. The cDNA was synthesized through reverse transcription of 1 μg of RNA using RevertAid First Strand cDNA Synthesis Kit (Thermo). qRT-PCR was performed using a 7500 sequence detection system (Applied Biosystems) and SYBR Green Realtime PCR Master Mix (Toyobo). GAPDH was used as internal control in qRT-PCR. Primer sequences for qRT-PCR are available in the in Supplementary Table 4. Relative mRNA expression was normalized to *GAPDH* and calculated by 2 ^−ΔCT^ method. Each experiment was performed in triplicate.

Shanghai Jiayin Biotechnology Co. Ltd performed sequencing and analysis on our RNA samples. TruSeq RNA preparation kit (Illumina) assembled the sequencing library, according to the manufacturer’s protocols. In Brief, Oligo (dT) magnetic beads were used to purify mRNA and fragment mRNA into short 200 bp fragments. Qubit 2.0 (Thermo) was used to assess the concentration of cDNA while Agilent 2100 Bioanalyzer was used to determine the length of library fragments. Finally, an Illumina HiSeq 2500 platform was used to perform RNA-sequencing. For bioinformatics analyses, we ran our raw sequence reads through FastQC (Babraham Institute, UK), and then we removed poor-quality reads and adapter sequences by using Cutadapt. HTSeq-count was used to determine read counts and R package, DESeq2 was used to determine differential gene expression.

### Chromatin Immunoprecipitation (ChIP) and ChIP-seq

ChIP assays were performed using the SimpleChIP Plus Sonication Chromatin IP Kit according to the manufacturer’s instructions (56383, Cell Signaling Technology) with modification [51]. Briefly, the HTR-8/SVneo cells were crosslinked with 1% formaldehyde (F8775, Sigma) for 10 min at room temperature. After sonication, the sheared chromatins were incubated with specific antibodies (anti-H3K4me3, ab6002, Abcam or anti-KDM5C, ab34718, Abcam); and then precipitated with Protein A or Protein G. The immunoprecipitated complex was washed, and the input and ChIPed DNA were purified using QIAquick PCR Purification Kit (Qiagen). Primer sequences used for ChIP-qPCR are listed in Supplementary Table 4.

Using bwa version 0.7.10 [52], ChIP-Seq data was aligned to the human genome (hg19) reference genome. By converting raw bam files to bigwig files using IGV tools [53], we could perform visualization of the read count data. For identifying peaks, MACS2 peak caller version 2.1.1 was used with an initial threshold *q*-value of 0.01 as the cutoff and the sonicated input as a control.

### CUT&Tag library generation and sequencing

CUT&Tag was performed with Hyperactive in situ ChIP Library Prep Kit (Vazyme Biotech, TD901) according to the manufacturer’s instructions. PCR was performed to amplify the libraries after extraction with phenol-chloroform and ethanol precipitation. All libraries were sequenced by Illumina Hi-Seq Xten.

### Statistical analysis

GraphPad Prism 6.0 software was used to statistically analyze our data. For most of our experimental data, a two-tailed Student’s *t*-test or the ANOVA test was used unless otherwise indicated. For clinical data, we utilized the nonparametric Mann–Whitney to test the expression of *KDM5C*, *TGFβ2*, and *RAGE* mRNA. Spearman’s rank correlation test was used to analyze correlation. Statistical significance was defined as having a *p* value of less than 0.05. Result values are shown as mean ± SEM of at least three independent experiments.

## Supplementary information

Supplementary information

Original Data File

Supplementary Table 1

Supplementary Table 2

Supplementary Table 3

Supplementary Table 4

## Data Availability

The accession number for the raw data of RNA-seq, ChIP-seq, and CUT&Tag reported in this paper is GEO: GSE141718 and GSE200540. All other relevant data are available from the corresponding author on request.
