# Supplementary material for: Elevated histone demethylase KDM5C increases recurrent miscarriage risk by preventing trophoblast proliferation and invasion
Source: Cell Death Discov. 2022 Dec 22;8:495. doi: 10.1038/s41420-022-01284-y (PMC9780362; doi:10.1038/s41420-022-01284-y)
Supplement: Supplementary file 1 — Supplementary information [file 41420_2022_1284_MOESM1_ESM.pdf]

Supplementary information

Elevated Histone demethylase KDM5C increases recurrent miscarriage risk by preventing trophoblast proliferation and invasion

Authors: Min Xiao, Yan Zheng, Meng-Xi Wang, Yi-Hua Sun, Juan Chen, Kang-Yong Zhu, Fan Zhang, Yun-Hui Tang, Fan Yang, Ting Zhou, Yue-Ping Zhang, Cai-Xia Lei, Xiao-Xi Sun, Shan-He Yu, Fu-Ju Tian

This supporting file includes:

Supplemental Materials and Methods

Supplemental Figures and Figure legends

Supplemental Materials and Methods

Flow cytometry analysis and cell sorting

For flow cytometric cell cycle analysis, Ki-67 staining (561126, BD Biosciences) were measured. For flow cytometric apoptosis analysis, Annexin V-APC/BV421 staining (550474/563973, BD Biosciences) and 7-AAD (559925, BD Biosciences) were measured. Flow cytometry data was collected using a BD Calibur and analyzed with FlowJo software. For the fluorescence-activated cell sorting (FACS), the cells were sorted out using a cell sorter (BD FACSAriaTM).

Immunohistochemistry

Immunohistochemical staining was performed. In brief, tissue slides were deparaffinized and rehydrated. Endogenous peroxidase activity was blocked with 0.3% hydrogen peroxide for 20 min. Slides were blocked with 5% FBS for 30 min and incubated with rabbit anti-KDM5C (OALA07572, Aviva Systems Biology, 1:100), or anti-TGFβ2 (ab36495, Abcam, 1:100), or anti-RAGE (ab3611, Abcam, 1:100) at 4°C overnight. After washing in PBS, the sections were incubated with biotinylated secondary antibodies and stained by using a Mouse and Rabbit Specific HRP/DAB IHC Detection Kit (ab236466, Abcam). Meyer's hematoxylin (Sigma-Aldrich) was used as a counterstain dye. A negative control was obtained by replacing the primary antibody with PBS. Images were captured with the Leica microscope (Leica, Buffalo Grove, IL).

Extravillous Explant assay

Small 2-3 mm villi explants were isolated from first-trimester human villi tissue (6-10 weeks of gestation) and then plated in 24-well culture dishes pre-coated with phenol red-free Matrigel® substrate (Corning Life Sciences) with DMEM/F12 media plus 10% FBS. Villi explant-initiated outgrowths were used for the subsequent experiments and are marked as 24 h. EVT migration from the distal end of the villi explants was recorded for 72 h. ImageJ Pro software was used to assess the distance of migration. To assess the regulation of KDM5C on the migration of EVTs, KDM5C siRNA (250 nM), or control siRNA (250 nM) was

transfected into villi explants from RM patients or HCs. The pictures were taken after 24 h and 72 h using Leica light microscope. Villi explants from HCs were treated with lenti-vector control or lenti-KDM5C lentivirus, and pictures after 24 h and 72 h of *in vitro* culture were obtained using Leica microscope. The following siRNA sequences were used in this study: KDM5C siRNA-1, sense, 5′-GCAGAGAAAUCGGGCAUUUTT-3′; KDM5C siRNA-1, antisense, 5′-AAAUGCCCGAUUUCUCUGCTT-3′; KDM5C siRNA-2, sense, 5′-CCUUUAAAGCU- GACUACUUTT-3′; KDM5C siRNA-2, antisense, 5′-AAGUAGUCAGCUUUAAAGG- TT-3′; KDM5C siRNA-3, sense, 5′-GCUGACACCUGAACUAUUUTT-3′; KDM5C siRNA-3, antisense, 5′-AAAUAGUUCAGGUGUCAGCTT-3′.

Western blotting

Total cell lysates were loaded onto 8%-12% SDS polyacrylamide gels for running and then transferred to PVDF membranes. After blocking, the membranes were incubated for 2 hr with the primary antibodies. After staining with horseradish perioxidase (HRP)-linked secondary antibodies, signal detection was performed using a chemiluminescence phototope-HRP Kit (Millipore). The following antibodies were used: anti-KDM5C (ab34718, Abcam, 1:500), anti-TGFβ2 (ab36495, Abcam, 1:1000), anti-RAGE (ab3611, Abcam, 1:1000) and anti-β-ACTIN (A5316, Sigma, 1:5000).

Colony formation assay

HTR-8 stable cell lines were seeded into six-well plates at a density of 8×10^2 cells/well. Cells were incubated at 37 °C in 5% CO_2 for about 10–14 days, fixed in 4% formalin for 15 minutes, and stained with Giemsa solution (AppliChem, Darmstadt, Germany) after two washes with PBS. The number of colonies was counted after cells were allowed to air dry at room temperature.

Cell proliferation assay

HTR8 cells were plated at a density of 2×10^3 cells per well in 96-well plates for the proliferation assay. Cell viability was analyzed at 24, 48 or 72 h using the CCK8 assay (Dojindo, Kumamoto, Japan).

Mouse trophoblast isolation

Percoll gradient Primary trophoblast were isolated by collagenase-DNase I digestion (Sigma-Aldrich, St. Louis, MO) and Percoll gradient (GE Healthcare, Amersham Place, UK) centrifugation from mature placenta on d 10.5, as previously described [6, 7]. The resultant trophoblast cell culture had a purity of approximately 80-90%, which was determined by immunostaining for cytokeratin 7. Purified trophoblasts were resuspend in culture medium (NCTC-135) for further experiments.

Supplemental Figures and Figure legends

Supplementary Figure 1

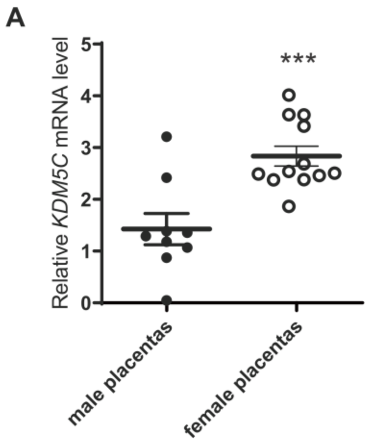

Supplementary Figure 1. KDM5C expression in male and female placentas.

(A) Level of *KDM5C* mRNA was determined in villi tissues of male placentas (n = 9) or female placentas (n = 11) of RM patients using qRT-PCR assay. Data are shown as the mean ± SEM; Student's *t* test was used to evaluate the statistical significance; ***$p <$ 0.001.

Supplementary Figure 2

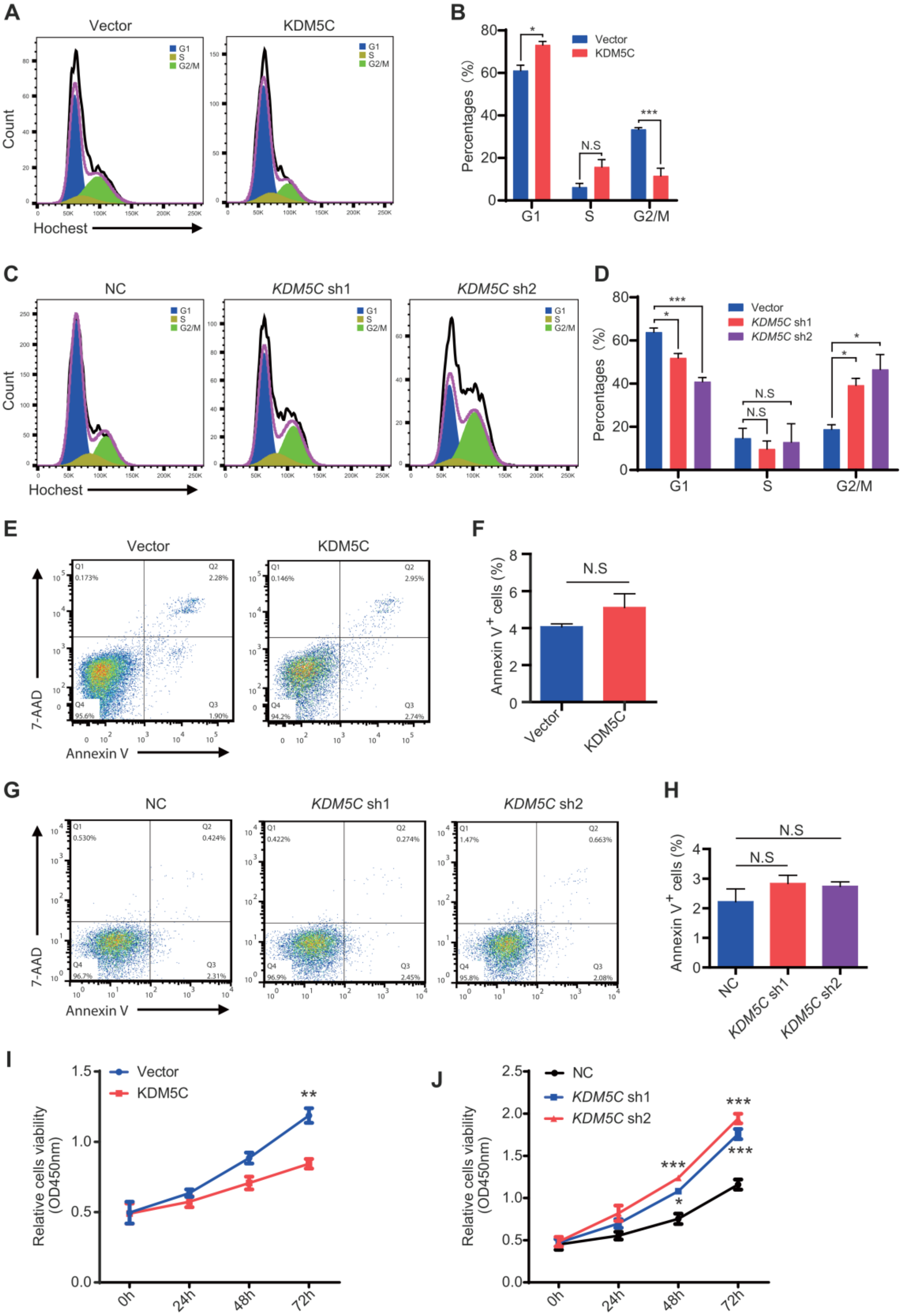

Supplementary Figure 2. KDM5C expression decreased trophoblast proliferation and invasion, related to Figure 2.

(A-D) Flow cytometric analyses of cell cycle and the percentages of G1, S, G2/M phase in HTR8 cells in HTR-8 cells stably transduced with vector control, or KDM5C-expressing vector (A-B), or in HTR-8 cells transduced with NC shRNA or two *KDM5C* shRNAs (C-D). (E-H) Flow cytometric analyses of Annexin V/7-AAD staining in HTR-8 cells stably transduced with vector control, or KDM5C-expressing vector (E-F), or in HTR-8 cells transduced with NC shRNA or two *KDM5C* shRNAs (G-H). (I-J) Cell proliferation was analyzed by the CCK8 assay in HTR-8 cells stably transduced with vector control, or KDM5C-expressing vector (I), or in HTR-8 cells transduced with NC shRNA or two *KDM5C* shRNAs (J). B, D, H, I, J, the ANOVA test; F, Student's t test. Data are shown as the mean ± SEM of three independent experiments; $*p < 0.05$, $**p < 0.01$, $***p < 0.001$.

Supplementary Figure 3

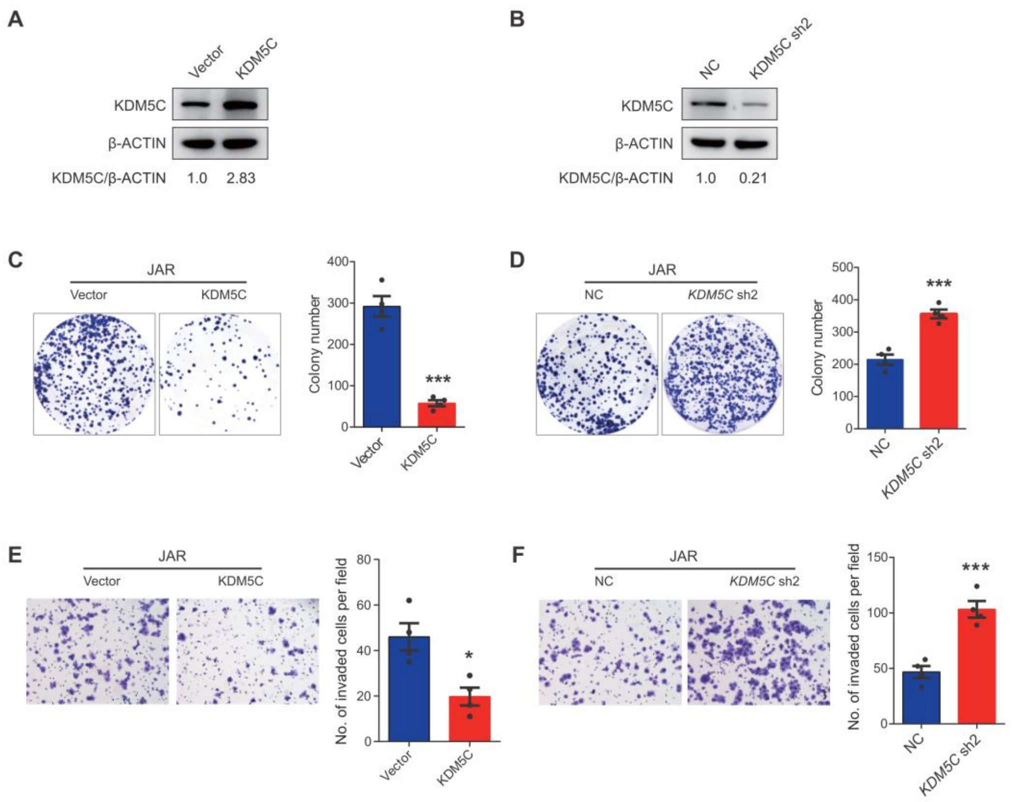

Supplementary Figure 3. KDM5C suppresses trophoblast proliferation and invasion of JAR cells, related to Figure 2.

(A, B) Western blotting assay of KDM5C protein of JAR cells stably transduced with vector control or KDM5C-expressing vector (A), or of JAR cells stably transduced with NC shRNA or *KDM5C* shRNA-2 (B). (C-D) Colony formation assay of JAR cells upon overexpression of KDM5C (C), or of JAR cells upon knockdown of *KDM5C* (D). (E-F) Cell invasion assay of JAR cells upon overexpression of KDM5C (E), or of JAR cells upon knockdown of *KDM5C* (F). Data are shown as the mean ± SEM of four independent experiments; Student's *t* test was used to evaluate the statistical significance; *$p < 0.05$, ***$p < 0.001$.

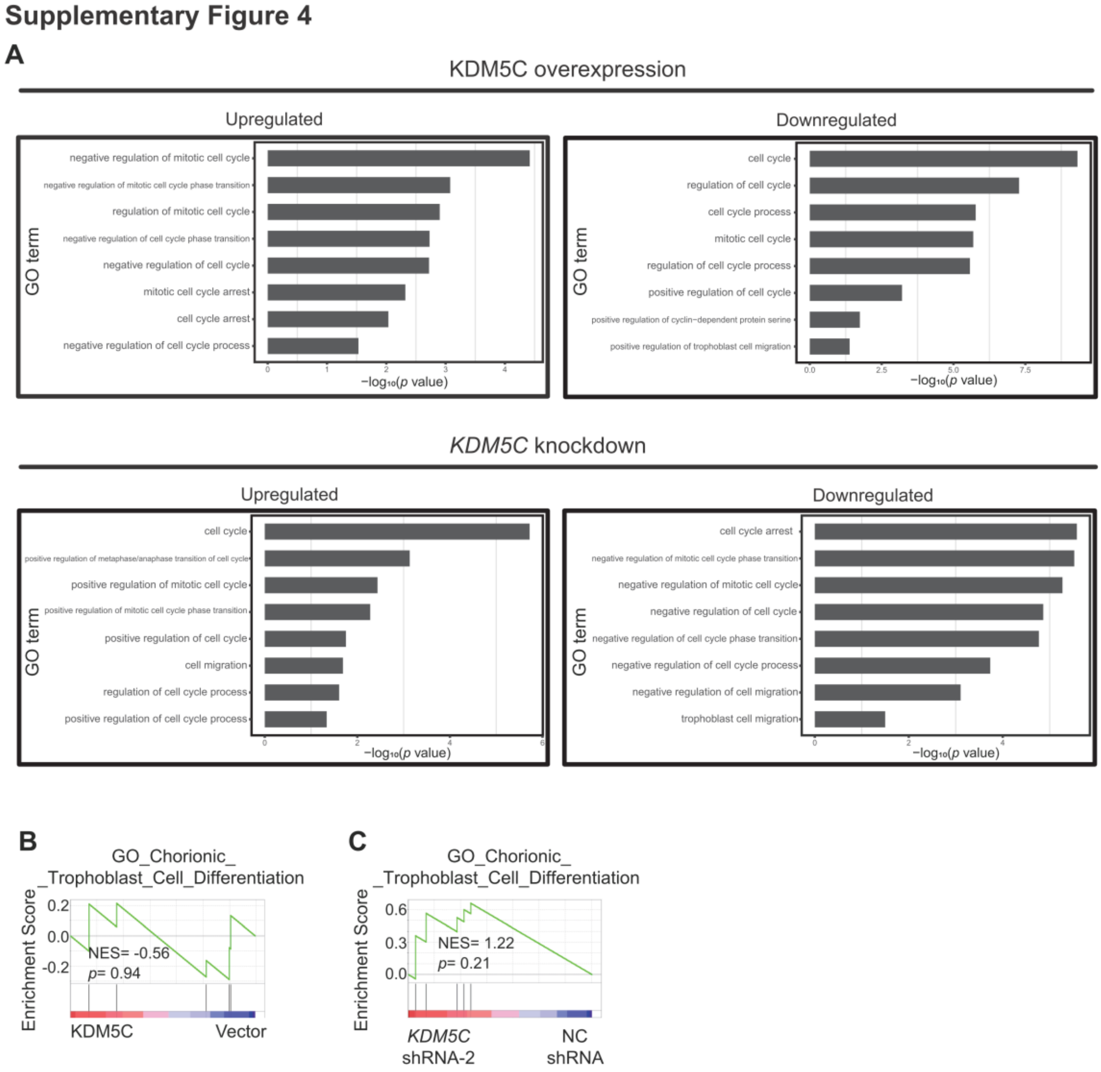

Supplementary Figure 4. KDM5C inhibits key gene ontology categories of trophoblast proliferation and invasion, related to Figure 4.

(A) Summary of the functional categories of genes significantly enriched in HTR-8 cells upon KDM5C overexpression (upper panel) or knockdown (bottom panel). Analyses were performed on the differentially expressed genes in HTR-8 cells by KDM5C overexpression or knockdown using DAVID. (B-C) GSEA of the expression profile of HTR-8 cells under KDM5C overexpression (B) or knockdown (C) using a chorionic trophoblast cell differentiation-associated signature.

Supplementary Figure 5

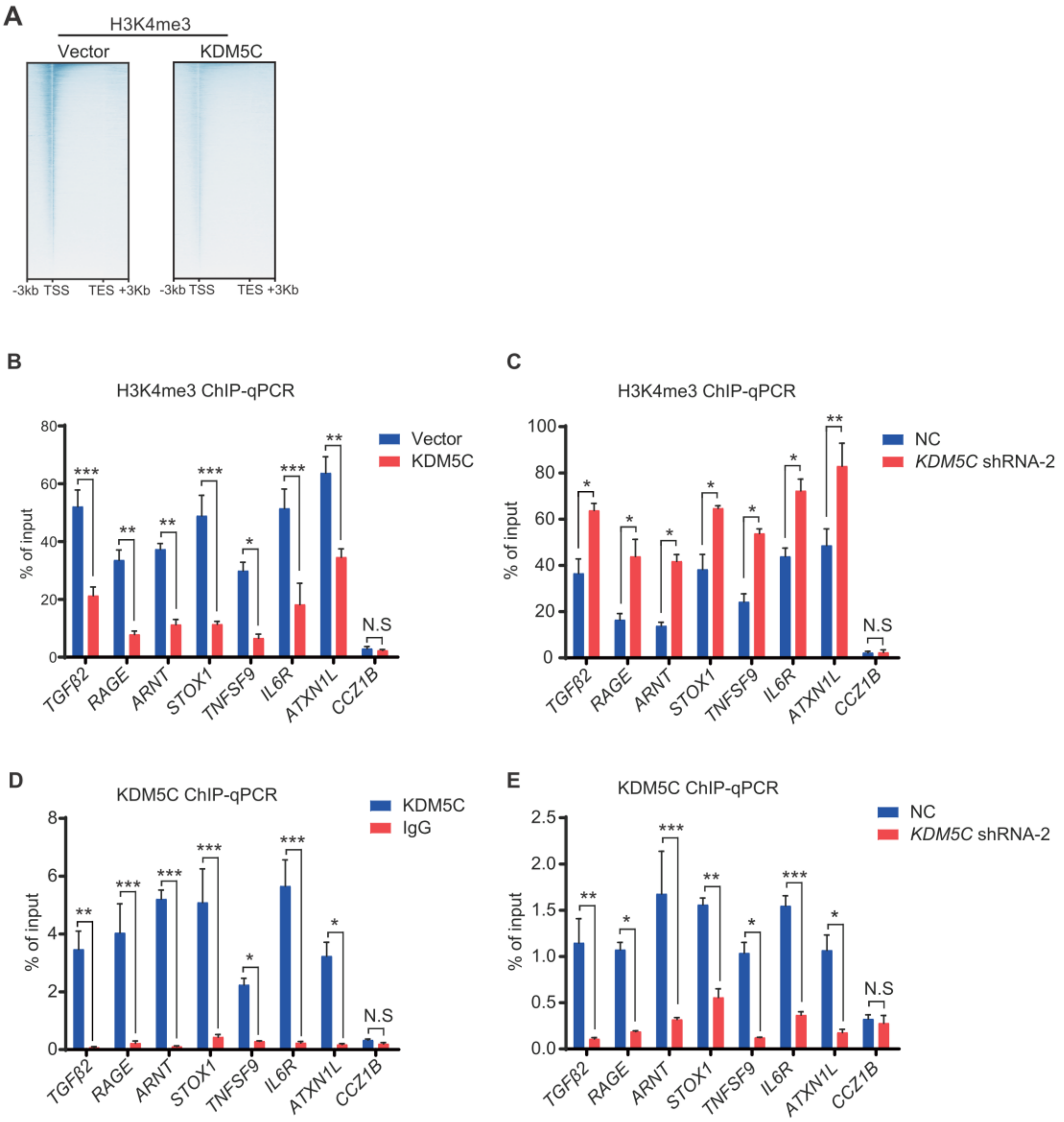

Supplementary Figure 5. KDM5C regulates dynamic H3K4me3 in the promoter of target genes, related to Figure 5.

(A) Heatmap of H3K4me3 ChIP-seq signal within ± 3kb genomic regions from anchors on the transcriptional start site (TSS) and transcriptional termination site (TTS) of HTR-8 cells transduced with control or KDM5C-expressing vector. (B, C) ChIP-qPCR assay of H3K4me3 occupancy at a number of gene loci in HTR-8 cells stably transduced with control or KDM5C-expressing vector (B), or in HTR-8 cells stably transduced with NC shRNA or *KDM5C* shRNA-2 (C). (D) ChIP-qPCR assay of KDM5C or IgG occupancy at a number of gene loci in HTR-8 cells stably transduced with KDM5C-expressing vector. (E) ChIP-qPCR assay of KDM5C

occupancy at a number of gene loci in HTR-8 cells stably transduced NC shRNA or *KDM5C* shRNA-2. Data are shown as the mean ± SEM of three independent experiments; the ANOVA test was used to evaluate the statistical significance; $*p < 0.05$, $**p < 0.01$, $***p < 0.001$.

Supplementary Figure 6

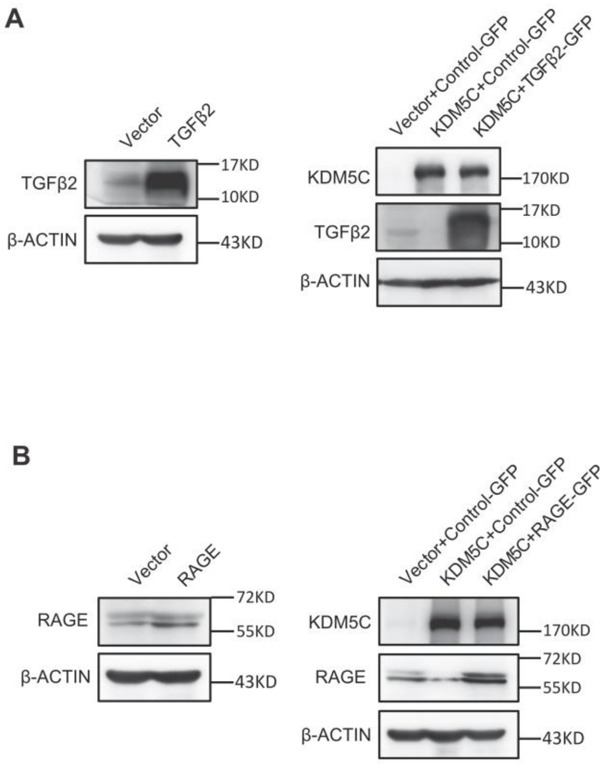

Supplementary Figure 6, western blotting assay of TGFβ2 or RAGE, related to Figure 6.

(A) Western blotting assay of TGFβ2 protein in HTR-8 cells transduced with vector control, or TGFβ2-expressing vector (left panel), western blotting assay of KDM5C and TGFβ2 protein for TGFβ2 overexpression experiments in HTR-8 cells transduced with control or KDM5C-expressing vector (right panel). (B) Western blotting assay of RAGE protein in HTR-8 cells transduced with vector control, or RAGE-expressing vector (left panel), western blotting assay of KDM5C and RAGE protein for RAGE overexpression experiments in HTR-8 cells transduced with control or KDM5C-expressing vector (right panel).
