# Supplementary material for: Elevated histone demethylase KDM5C increases recurrent miscarriage risk by preventing trophoblast proliferation and invasion
Source: Cell Death Discov. 2022 Dec 22;8:495. doi: 10.1038/s41420-022-01284-y (PMC9780362; doi:10.1038/s41420-022-01284-y)
Supplement: Supplementary file 2 — Original Data File [file 41420_2022_1284_MOESM2_ESM.pdf]

Figure 1C

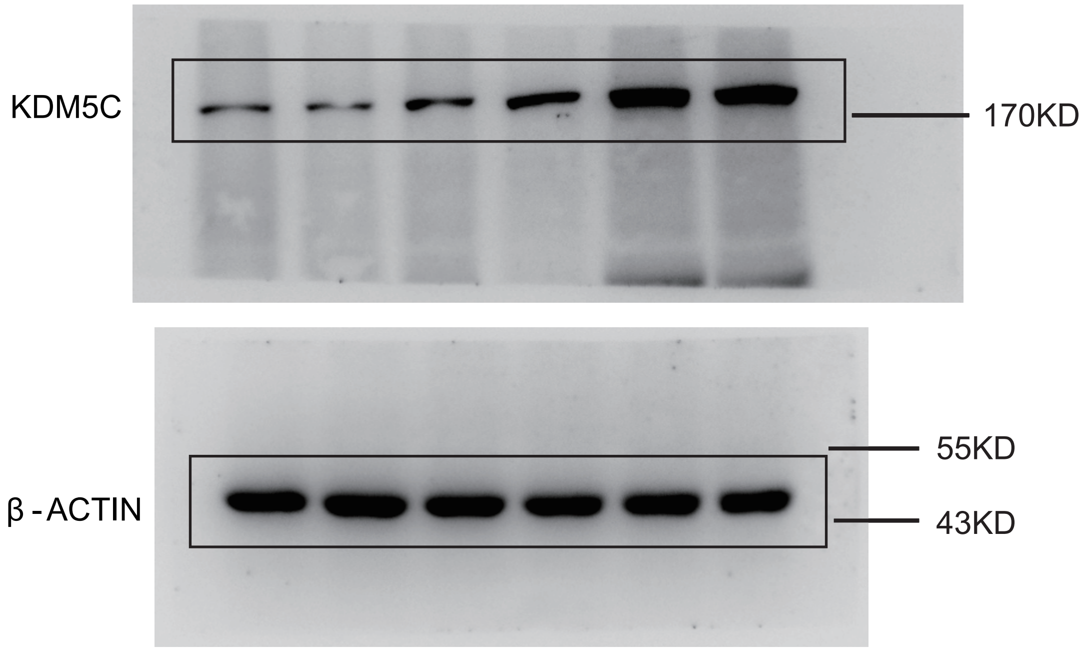

Figure 2A, right panel

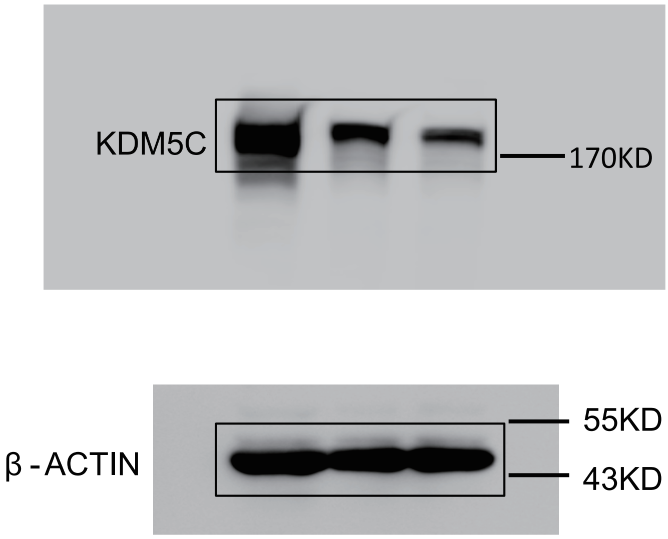

Supplementary Figure. The uncropped scans of blots

Figure 2A, left panel

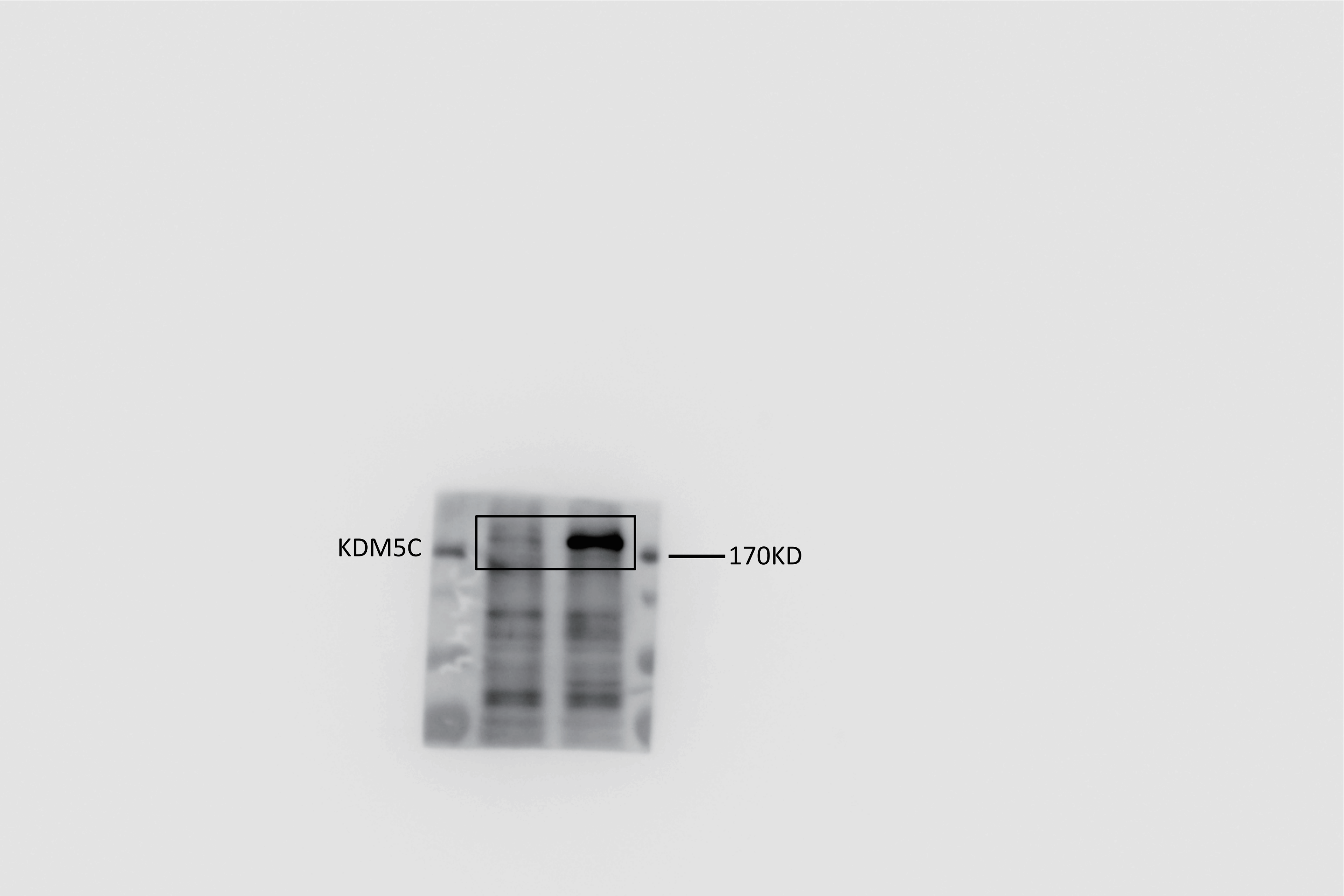

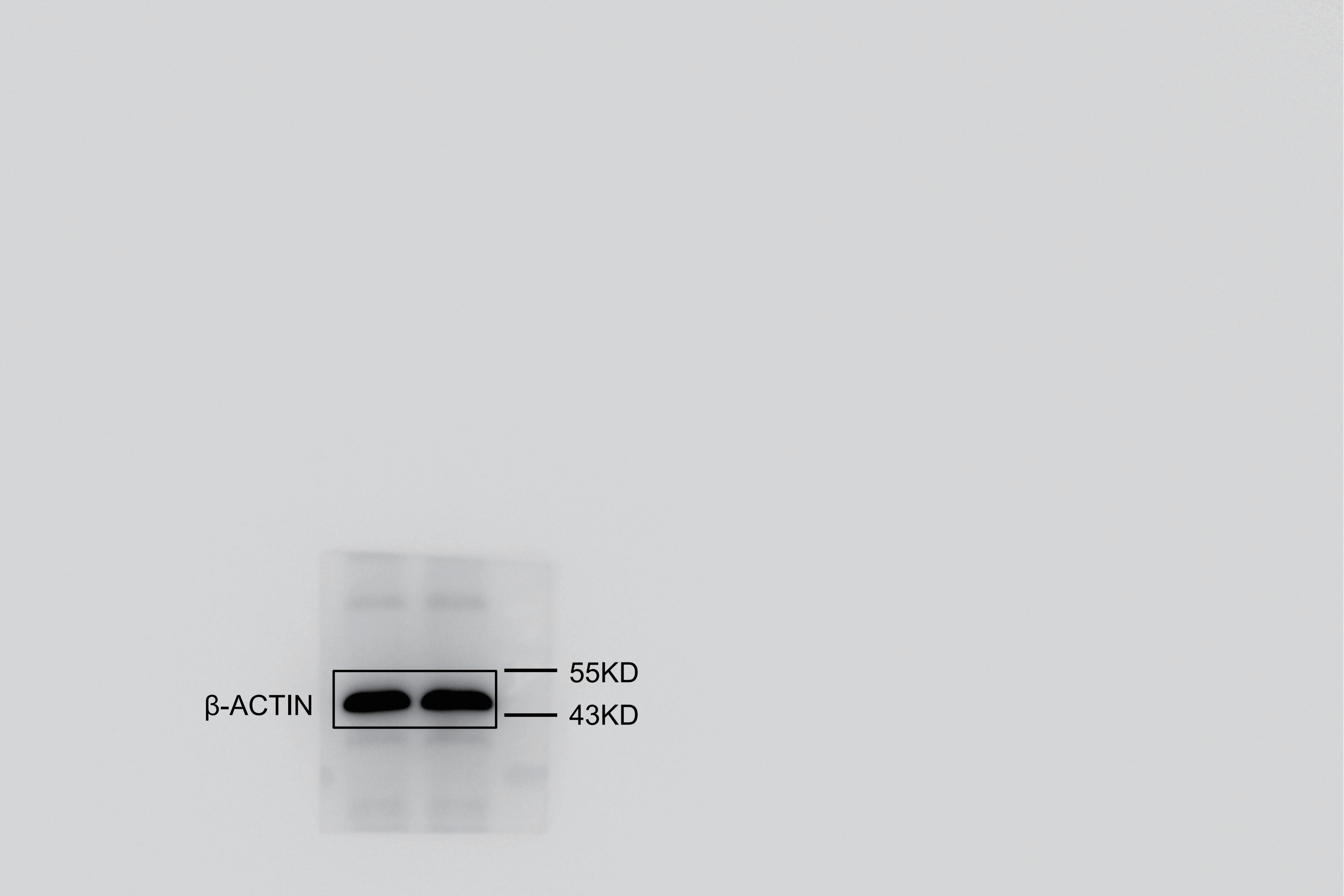

Supplementary Figure continued

Figure 3A

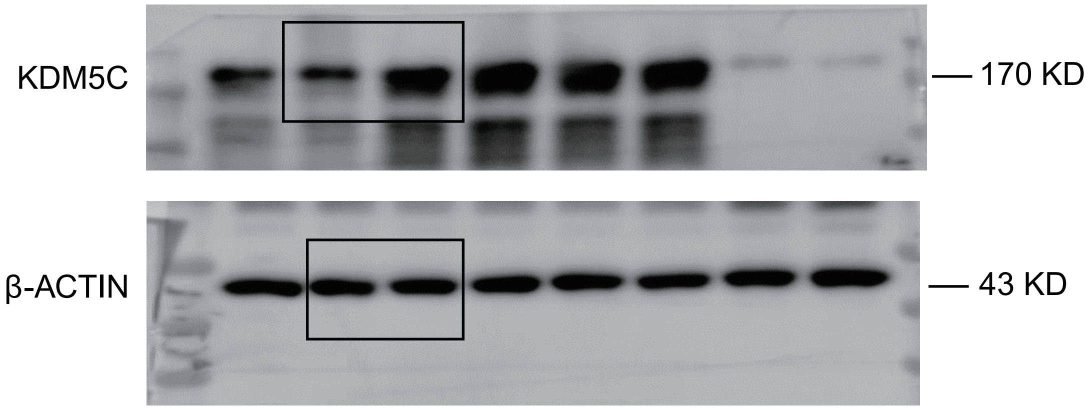

Figure 3B

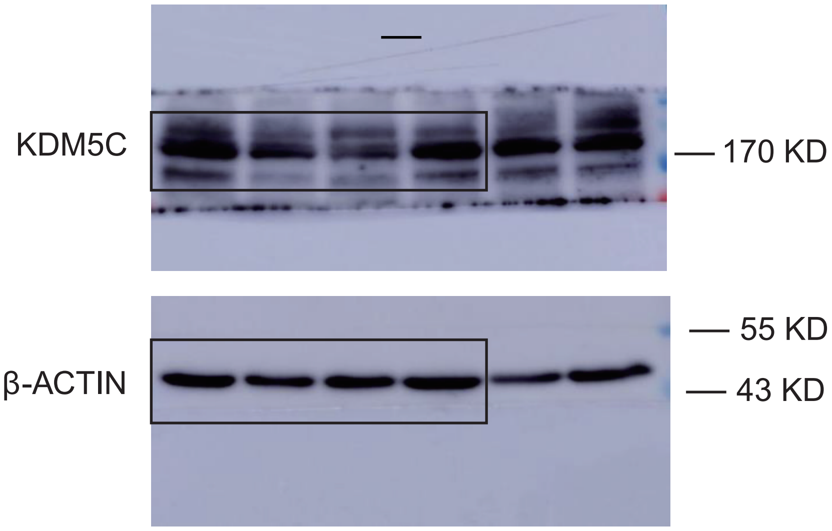

Figure 3C

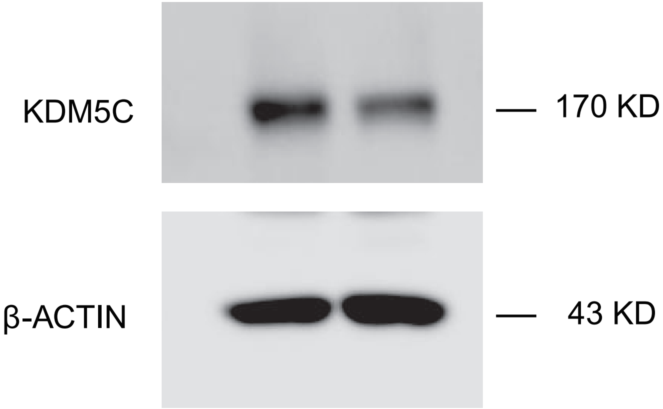

Supplementary Figure continued

Figure 8B

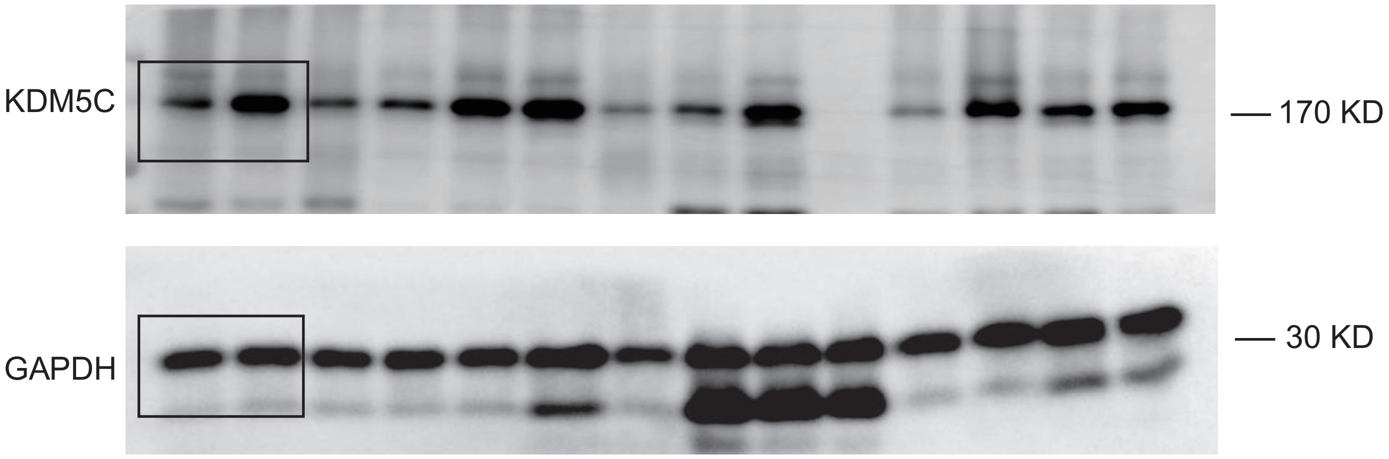

Figure 8D

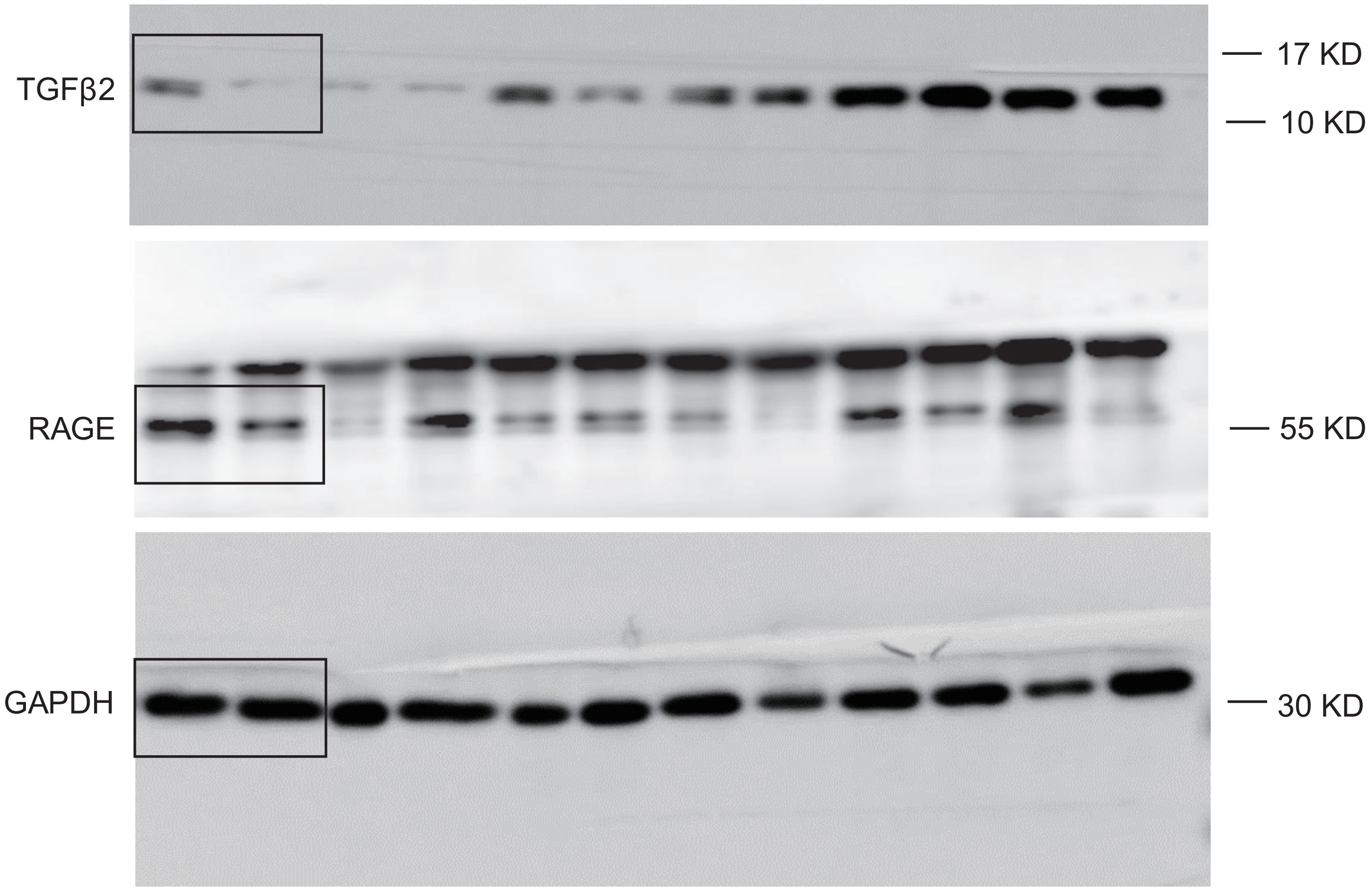

Supplementary Figure continued

Figure 8E

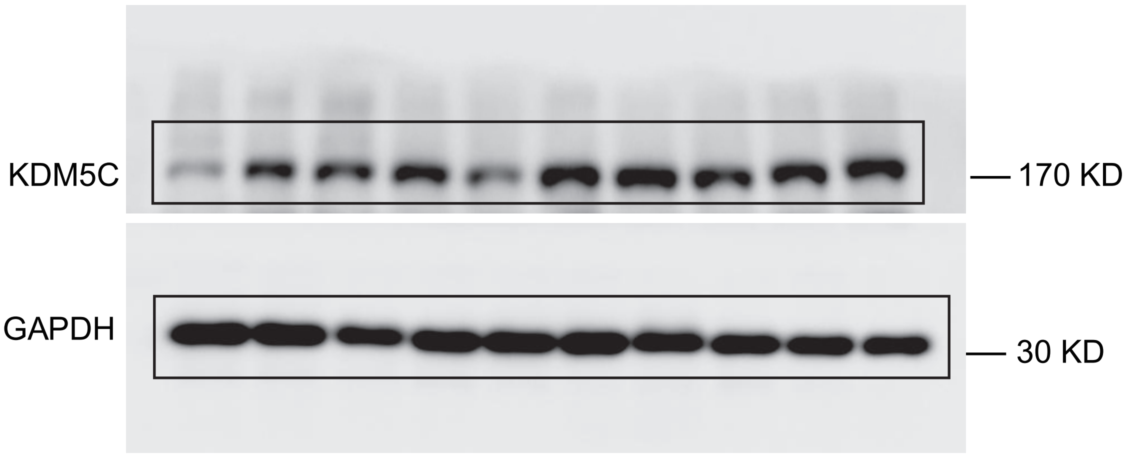

Figure 8H

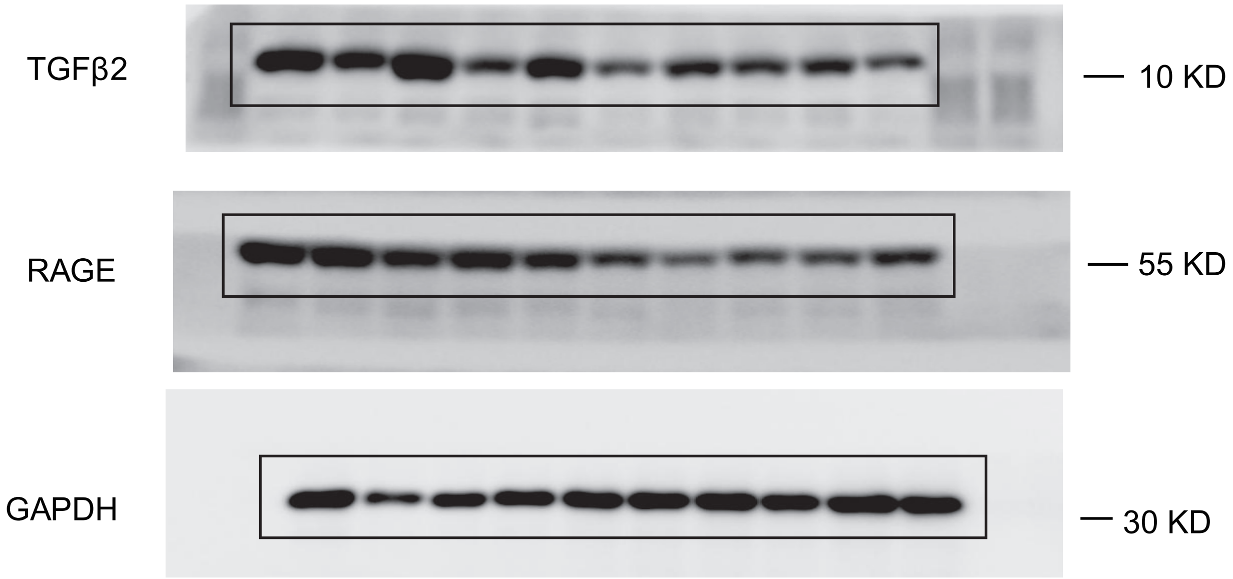

Suplementary Figure 3A

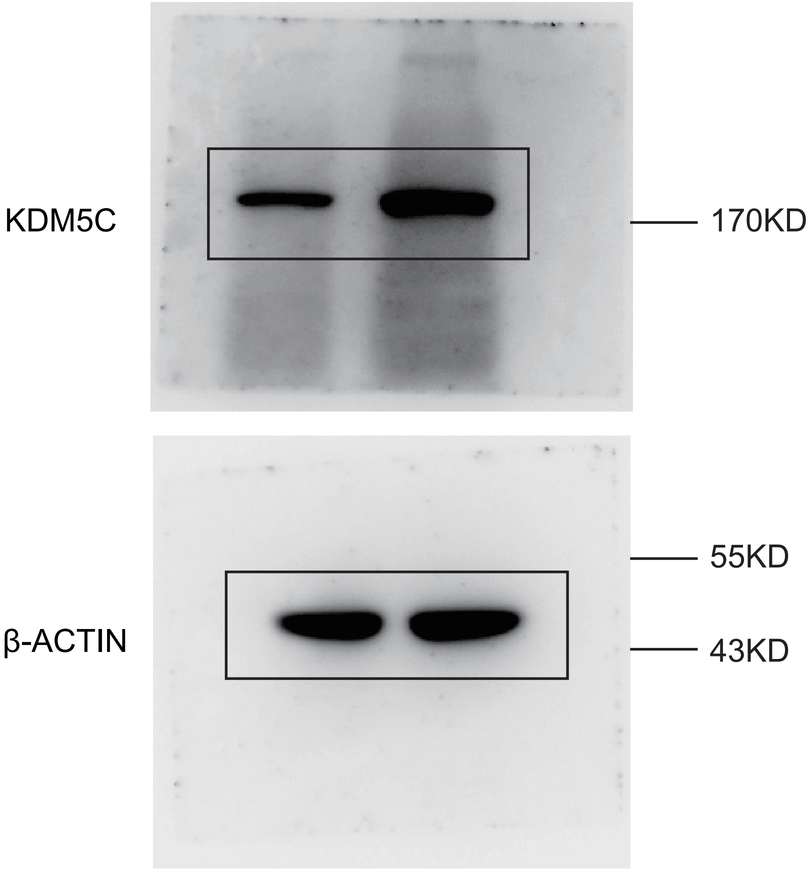

Suplementary Figure 3B

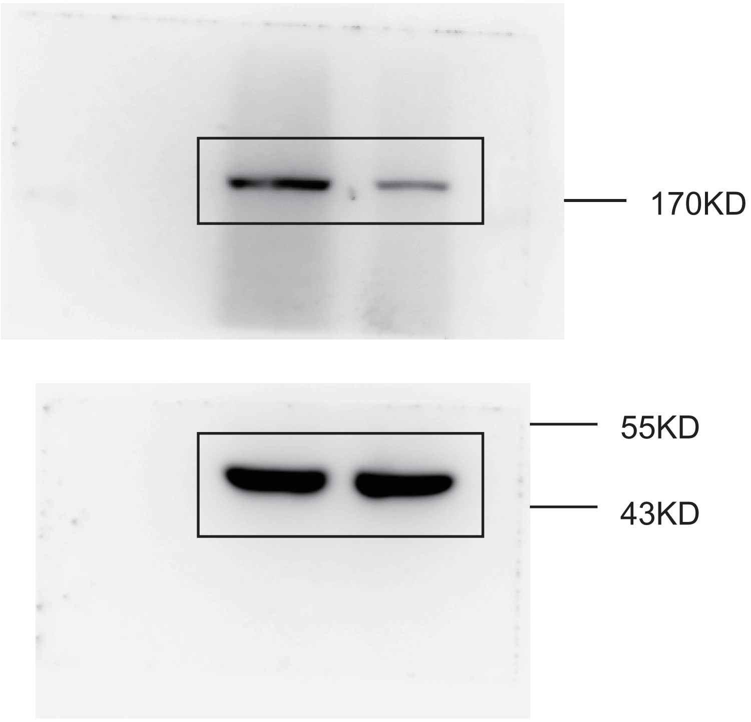

Suplementary Figure 6A

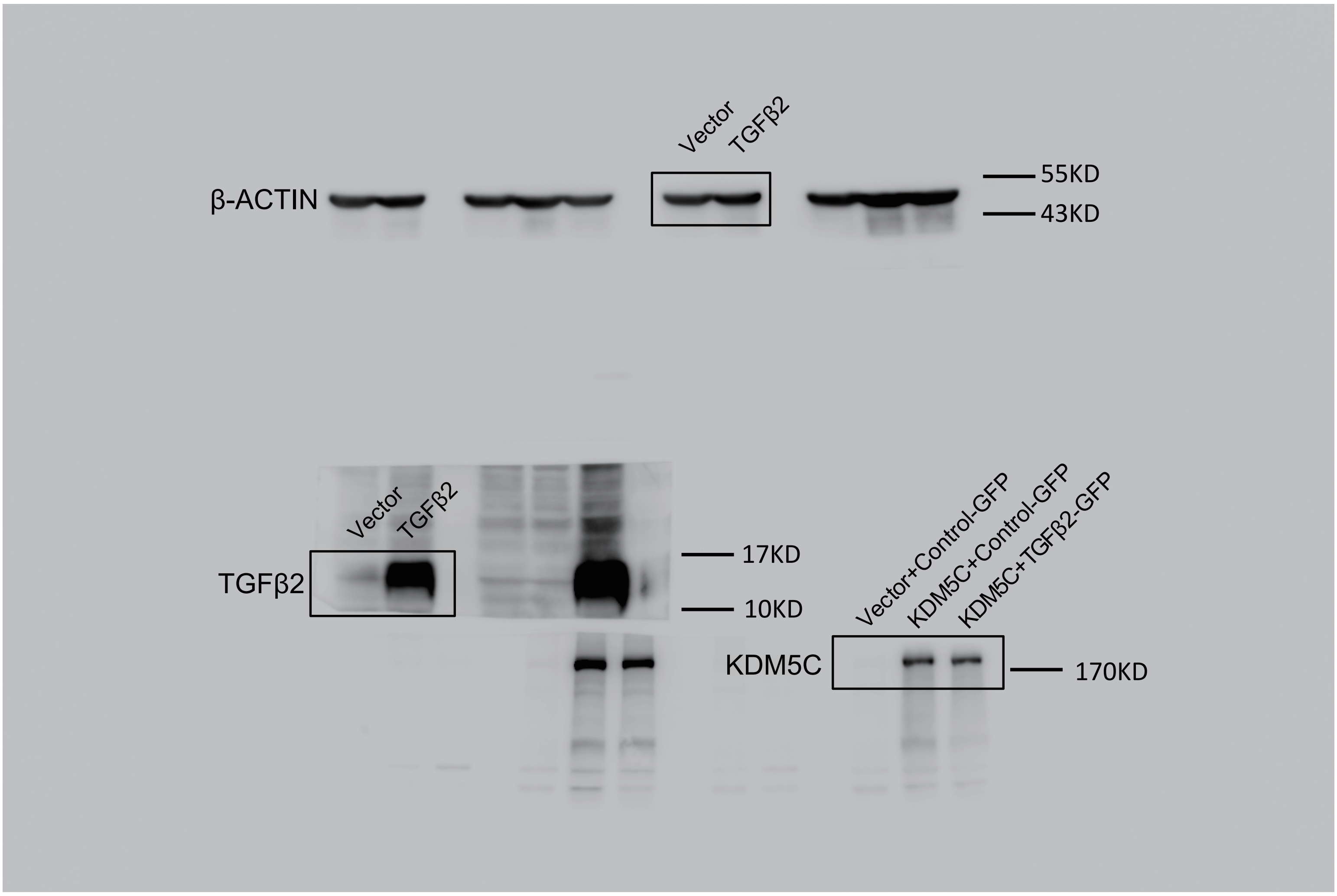

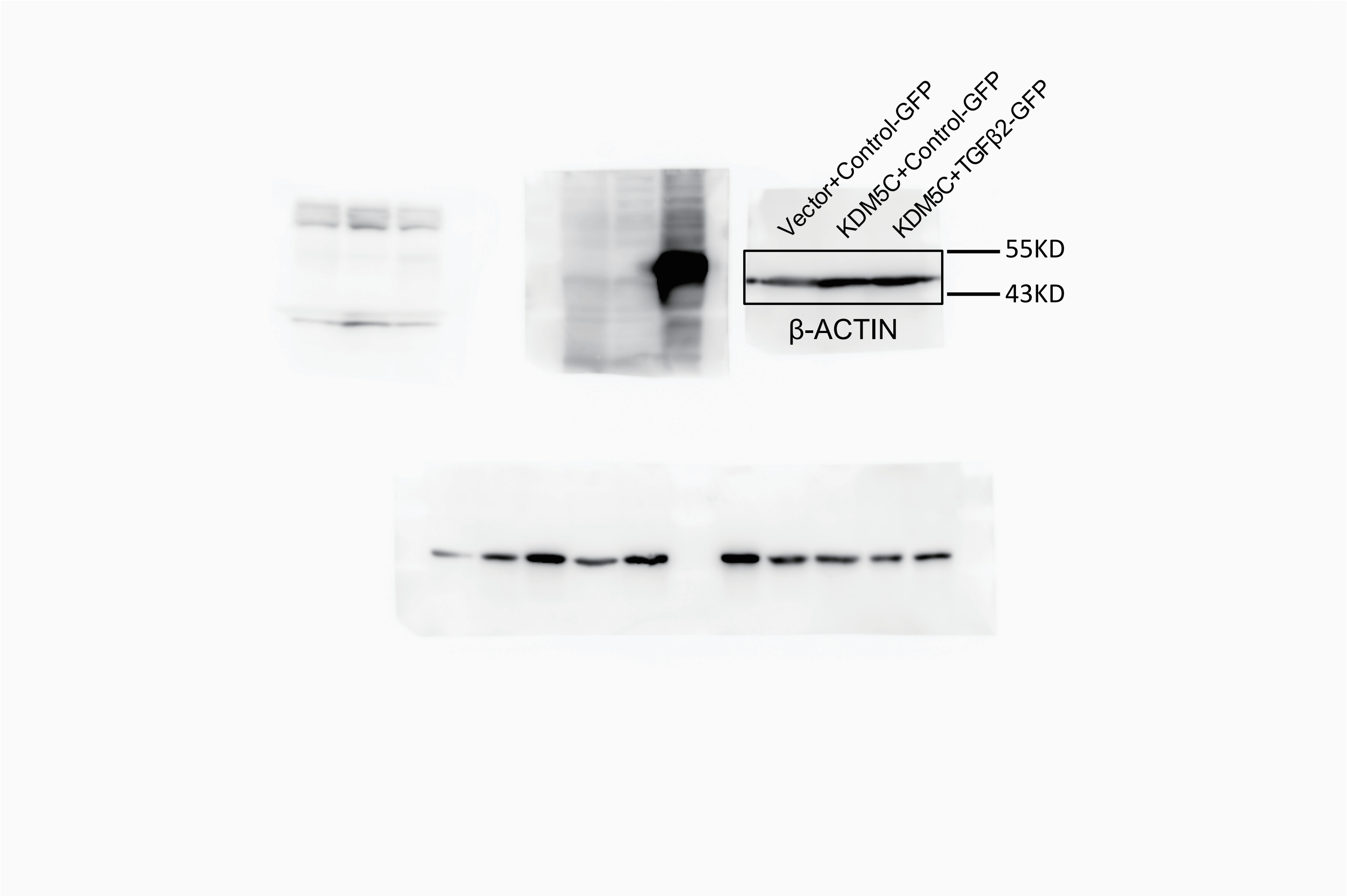

Supplementary Figure continued

Suplementary Figure 6A

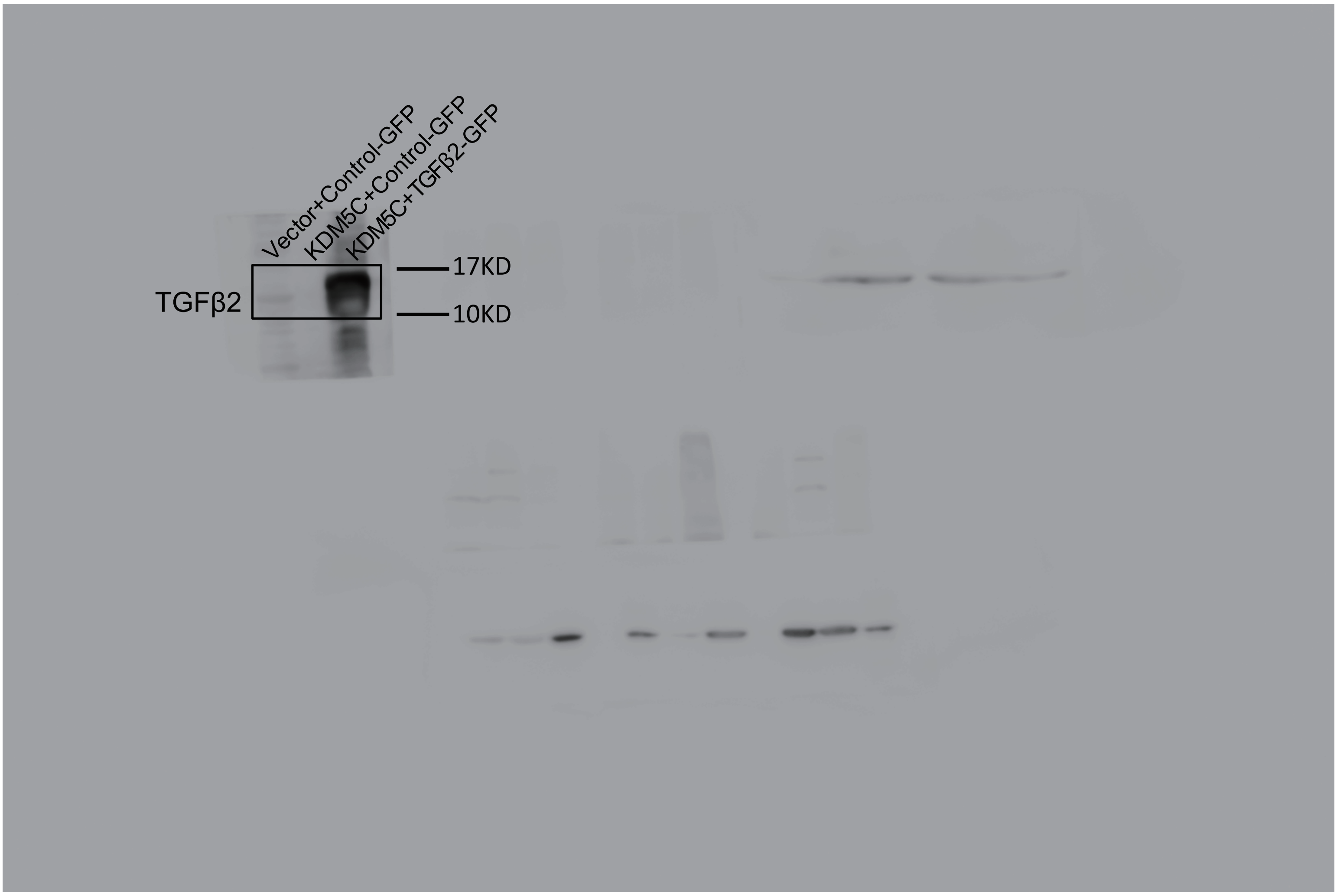

Suplementary Figure 6B

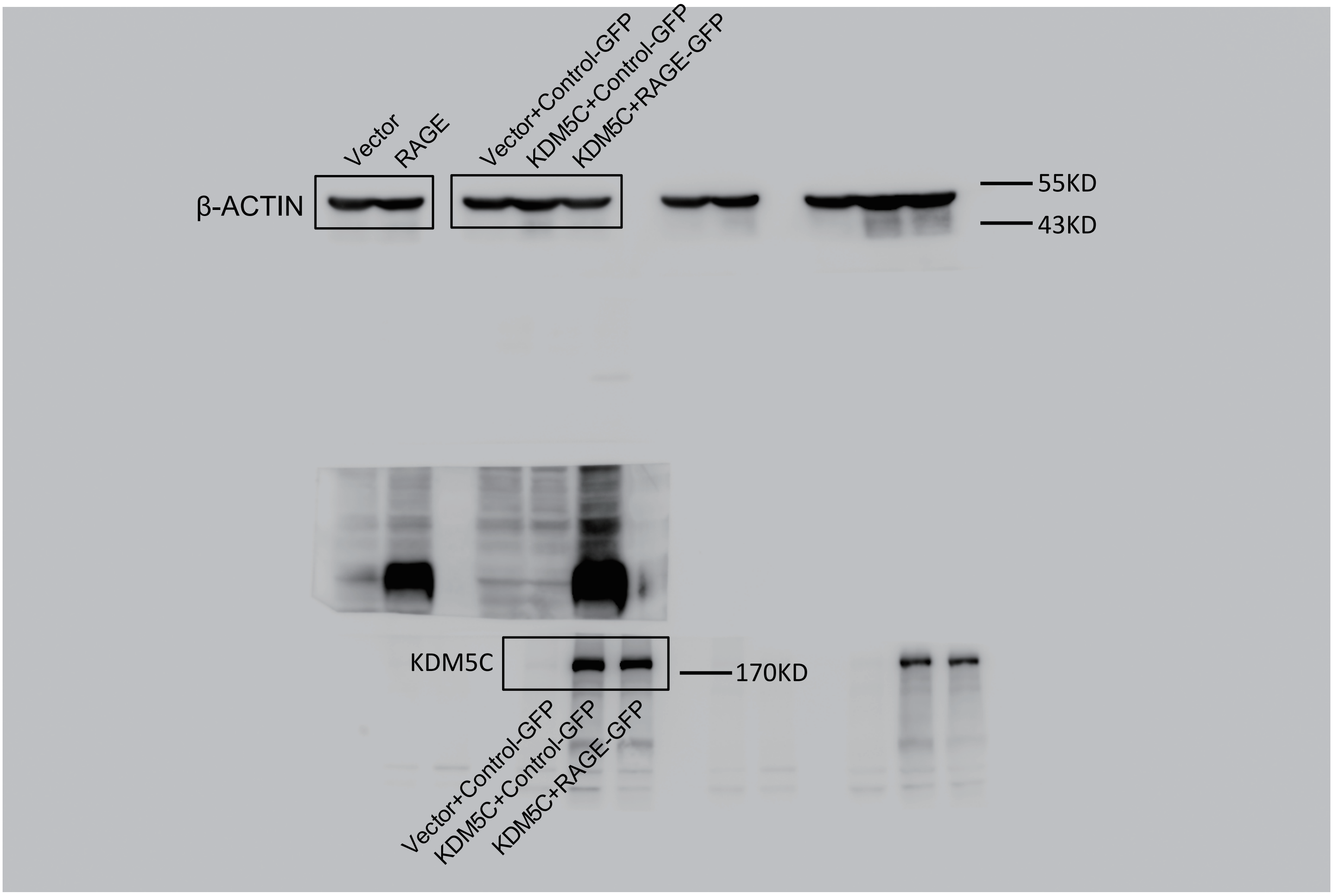

Supplementary Figure continued

Suplementary Figure 6B

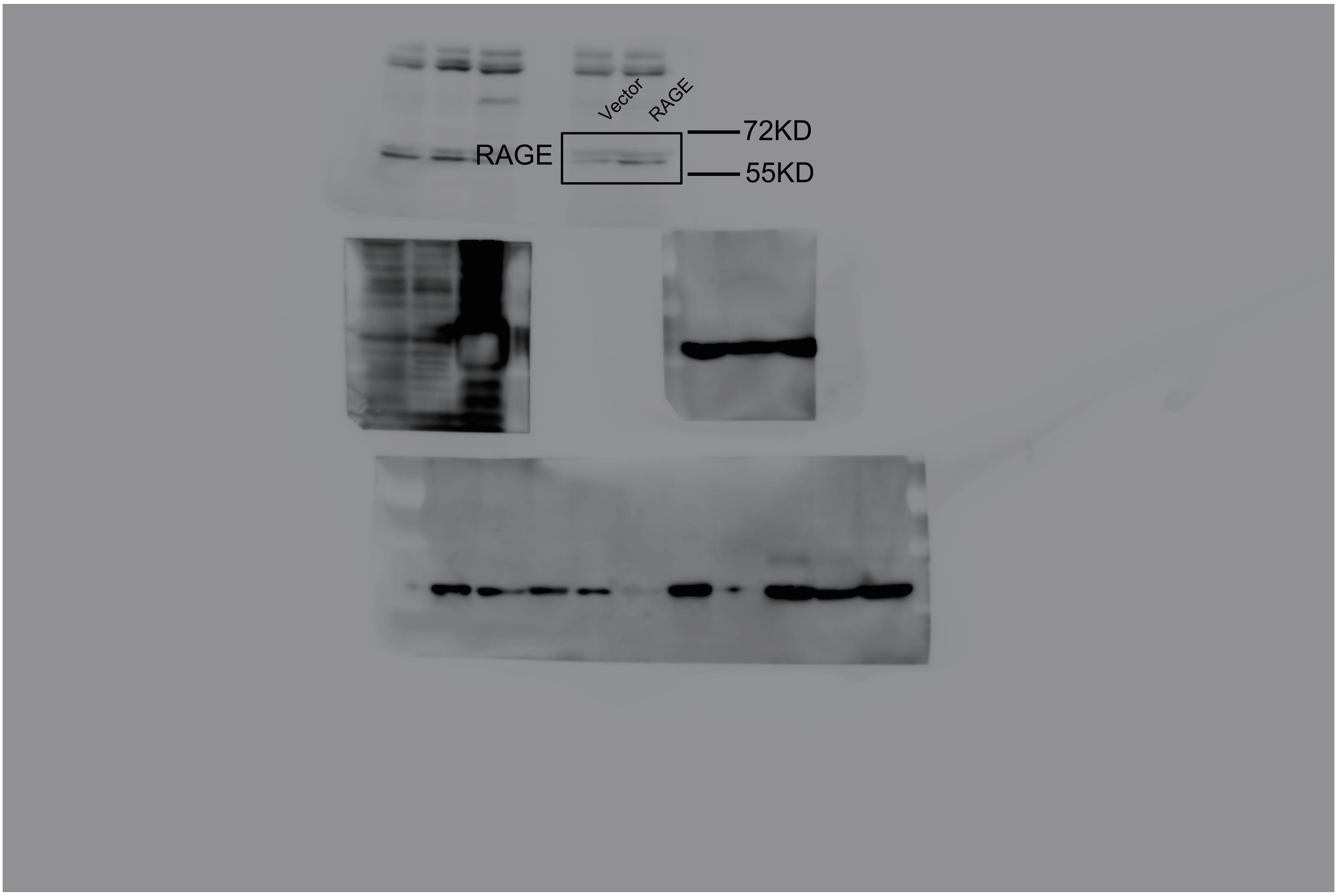

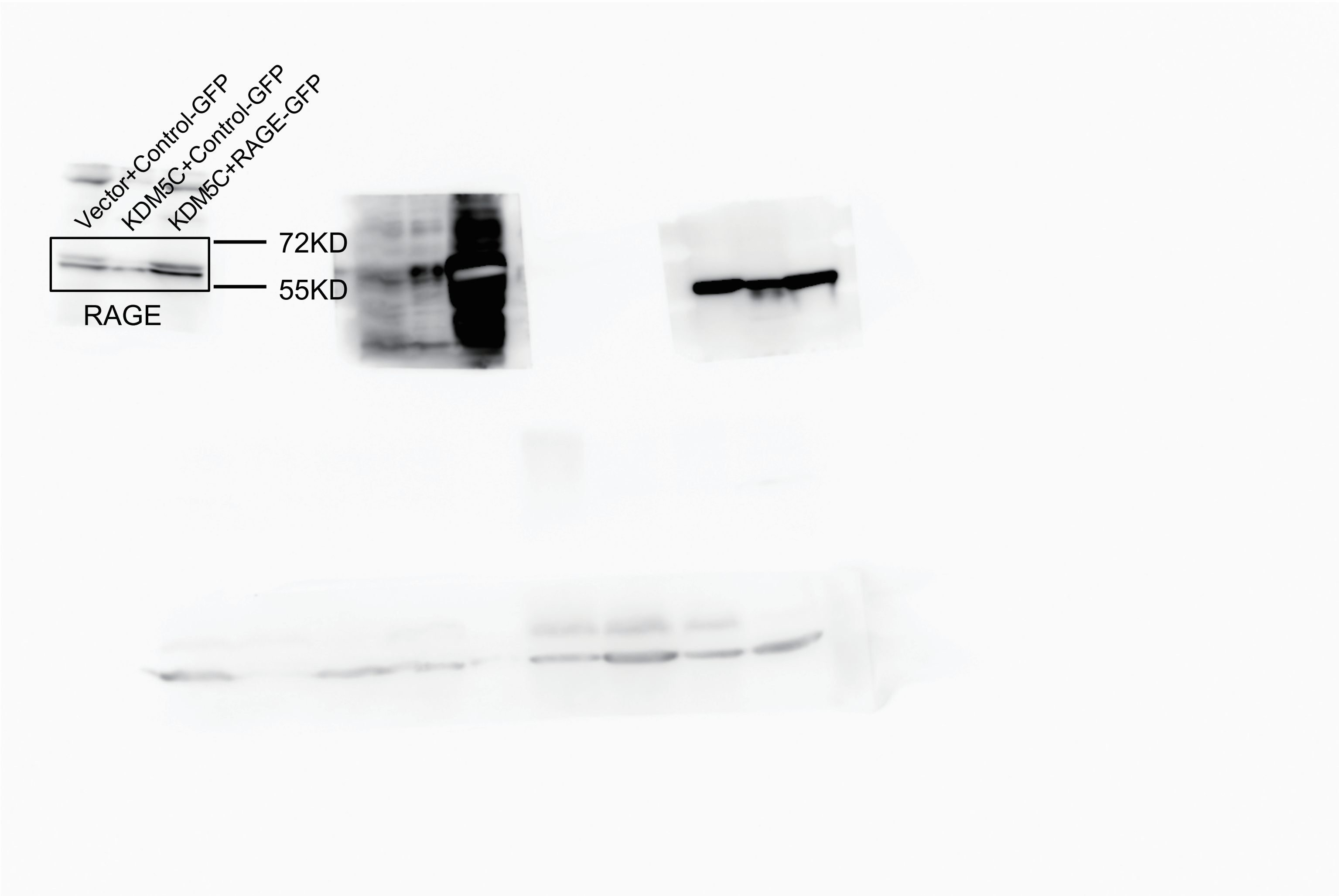

Supplementary Figure continued
