# Supplementary material for: Elevated histone demethylase KDM5C increases recurrent miscarriage risk by preventing trophoblast proliferation and invasion
Source: Cell Death Discov. 2022 Dec 22;8:495. doi: 10.1038/s41420-022-01284-y (PMC9780362; doi:10.1038/s41420-022-01284-y)
Supplement: Supplementary file 5 — Supplementary Table 3 [file 41420_2022_1284_MOESM5_ESM.pdf]

Supplementary Table 3. Clinical and demographic characteristics of the patient groups in the screening and validation studies

	Type of samples	Maternal age (years)	Previous pregnancy losses	Gestation-parturition	Gestational age at sampling (days)	Karyotype
1	HC	31	0	G3P2	53	46,XY
2	HC	43	0	G3P1	47	46,XX
3	HC	25	0	G1P0	46	46,XY
4	HC	40	1	G4P1	49	46,XX
5	HC	28	0	G2P1	65	46,XY
6	HC	40	0	G3P2	44	46,XY
7	HC	19	0	G4P0	53	46,XX
8	HC	20	0	G1P0	50	46,XX
9	HC	27	0	G2P1	49	46,XX
10	HC	25	0	G2P1	42	46,XY
11	HC	19	0	G3P1	41	46,XY
12	HC	46	0	G1P0	53	46,XY
13	HC	25	0	G1P0	46	46,XX
14	HC	24	0	G2P1	45	46,XX
15	HC	18	0	G4P2	49	46,XY
16	HC	29	1	G4P1	50	46,XX
17	HC	33	0	G2P1	50	46,XY
18	HC	31	0	G1P0	44	46,XY
19	HC	29	0	G4P1	40	46,XX
20	HC	27	0	G4P0	35	46,XX
21	HC	39	0	G3P1	43	46,XY
22	HC	33	0	G1P0	43	46,XY
23	HC	32	0	G2P1	43	46,XX
24	HC	22	0	G1P0	49	46,XY
25	HC	26	0	G2P0	65	46,XY
26	HC	33	0	G2P1	53	46,XX
27	RM	26	2	G3P0	54	46,XX
28	RM	27	2	G3P0	51	46,XY
29	RM	26	3	G4P0	63	46,XX
30	RM	35	2	G3P0	53	46,XX
31	RM	32	2	G3P0	48	46,XY
32	RM	36	2	G3P0	44	46,XY
33	RM	29	3	G4P0	43	46,XY
34	RM	33	3	G4P0	61	46,XY
35	RM	31	2	G3P0	56	46,XX
36	RM	29	2	G3P0	49	46,XX
37	RM	35	2	G3P0	42	46,XY
38	RM	42	2	G3P0	45	46,XY
39	RM	27	3	G4P0	47	46,XX
40	RM	27	4	G5P0	40	46,XX
41	RM	30	2	G3P0	52	46,XX

42	RM	28	2	G3P0	49	46,XX
43	RM	35	3	G5P1	54	46,XY
44	RM	29	4	G5P0	43	46,XY
45	RM	35	3	G4P0	43	46,XX
46	RM	32	2	G3P0	48	46,XY
47	RM	34	3	G5P1	42	46,XY
48	RM	34	4	G5P0	40	46,XX
49	RM	30	3	G4P0	47	46,XX
50	RM	31	2	G3P0	43	46,XY
51	RM	31	3	G4P0	48	46,XY
52	RM	35	2	G4P1	42	46,XX
53	RM	35	4	G5P0	62	46,XY
54	RM	27	2	G3P0	49	46,XY
55	RM	30	2	G3P0	44	46,XX
56	RM	37	4	G5P0	56	46,XX
57	RM	43	3	G4P0	55	46,XX

G, for gestation which means pregnancy; P, for production which means production. For example, G2P1 means two pregnancies and one production.
